# GPT-4 shows comparable performance to human examiners in ranking open-text answers

**DOI:** 10.1038/s41598-025-21572-8

**Published:** 2025-10-08

**Authors:** Abdullah Al Zubaer, Michael Granitzer, Stephan Geschwind, Johann Graf Lambsdorff, Deborah Voss

**Affiliations:** 1 Department of Computer Science and Mathematics, University of Passau, Passau, Germany; 2 Department of Business Administration, Economics and Business Information Systems, University of Passau, Passau, Germany

**Keywords:** Automated grading systems, Examination tasks, GPT-4, Inter-rater reliability, Open-text answers, Computer science, Information technology

## Abstract

Can GPT-4 replace human examiners? To address this question we explore the performance of GPT-4 as an examiner of answers to open-text questions. We formulate questions and sample solutions in the field of macroeconomics and collect answers from cohorts of undergraduate students. We then conduct a fair competition between GPT-4 and human experts, employing their expertise to assess the quality of the answers. We observe that the substitution of GPT-4 for a human examiner does not decrease inter-rater reliability on tasks that rank the quality of answers. We run checks on potential biases (whether GPT-4 prefers AI-generated or lengthy answers). We find no consistent evidence of such biases. Our findings are robust to tilting the competition to one side’s advantage, by using inferior or advanced prompting strategies. Our results are more attenuated on tasks where GPT-4 assigns points to student answers. Here, GPT-4 shows a bias towards longer answers. Overall, our study cautiously supports the utilization of GPT-4 as an assistant for automated grading systems, particularly those where answers are ranked according to their quality.

## Introduction

Generative AI (Artificial Intelligence) models, such as ChatGPT^1^, have the potential for broad repercussions on society in general and the educational system in particular, owing to their ability to produce high quality, human-like conversation. The fourth version of GPT (Generative Pre-trained Transformer)^2^, launched in March 2023, is especially recognized for its capacity to perform ‘in-context learning’^3^. It can generate responses based on a prompt containing examples or instructions to perform specific tasks relying entirely on its linguistic capability^4^. It is therefore highly adaptable in various scenarios despite being a generalized language model. In education, it might be used by students to complete assignments or write papers, but also by teachers to assess and grade them. While the former poses challenges to common examination practices^5^, the latter presents an opportunity to reduce the workload of time-constrained teaching staff^6^. As to explore this opportunity, this study investigates GPT-4’s capabilities in grading short open-text answers in the field of macroeconomics.

In the role of a student, GPT has been shown to perform remarkably well on single- and multiple-choice questions in micro- and macroeconomics, even outperforming many students and ranking in the top 1% in macroeconomics^7^. This finding is consistent with a wide range of evidence across different types of questions, subject areas, and levels: It includes single- and multiple-choice questions in medicine^8–10^ and law^11^, but also more complex essay-writing tasks. For instance, GPT has been shown to surpass high-school English essays^12^ as well as undergraduate biology essays^13^, and to perform on par with second-year university students in physics^5^. Thus, despite some studies finding weaker performances compared to humans (for instance, Martínez^14^ for essay questions in law) or cautioning that it occasionally exhibits behaviors indicative of plagiarism and hallucination^13^, the general tone of this literature is rather positive towards GPT’s capabilities to perform similarly well to students in various domains and at different levels. This is true even outside of English-speaking contexts, such as Portuguese^15^, German^16,17^, Spanish^18^ or Polish^19^. While being a good student might make it more likely to become a good teacher, the two are obviously separate questions. Our study rather adds to the literature on the latter question of being a good teacher.

When it comes to using GPT as a teacher, recent studies suggest a positive effect of using generative AI to assist teaching activities^20–22^. Taking a step further, we investigate the potential of GPT to replace – rather than merely assist – teaching activities, in particular as an examiner of short open-text answers. In this, we are more closely aligned with research on the performance of AI as compared to human teachers: A few recent studies have conducted experiments similar to ours in that they have generative AI evaluate student answers based on a prompt that includes the assignment, the student submission, grading criteria and instructions on how to grade^23–26^. They often find a positive correlation between scores assigned by generative AI and human examiners^23,26^, interpreting this as a satisfactory performance of the former. A limitation to these studies is that they do not always provide a level playing field for comparing humans and AI. In particular, if a generative AI is superior to humans, this would remain undetected, as these studies generally take the human assessment as the ground truth. A superior performance by AI induces an imperfect correlation, which is erroneously interpreted as an inferior performance. In fact, there exist many reasons for human fallibility that might not be relevant to a generative AI including subjective bias, cognitive limitations, fatigue, or inattention. We differ from this literature by allowing our human examiners to be fallible and using test methods that might also detect the superiority of a generative AI.

Tate et al.^25^ is, to the best of our knowledge, the only other study that has the capacity to detect a superiority of generative AI compared to human raters. Comparing inter-rater reliability (IRR) between teams of two humans and human-AI teams, they find that GPT-3.5 performs inferior to humans as IRR drops when replacing a human examiner with GPT. Ratings are point-based assessments where raters are asked to score answers from 1 to 6. Similar to Nilsson and Tuvstead^23^ and Sessler et al.^26^, their results show that GPT differs from human raters in the underlying distribution of points awarded, in particular due to its tendency to never award minimum or maximum points. If GPT’s performance is inferior mainly due to this difference, its performance might be improved by holding the distribution constant across different types of raters. A potential method that achieves this is by ranking student answers relative to each other instead of assigning points to them individually.

Our contribution to the existing literature is thus twofold: First, this study systematically compares the performance of human examiners and GPT-4 on a level playing field. We achieve this by distinguishing between the role of the teacher, who provides a sample solution to an open-text question and instructions on how to assess student answers, and that of the raters, who assess the answers. This allows us to use fallible human examiners and GPT-4 as our raters, provide them with identical instructions and compare their subsequent performance. Second, we employ two methods to arrive at such comparisons, one based on absolute points awarded for student answers and another on relative ranks. We acknowledge that absolute scoring, where each response is evaluated independently against a rubric, is more common in most educational settings. However, relative ranking also plays a role in practice, particularly when “grade curves” are applied that enforce fixed grade distributions (e.g. assigning an A to the top 20%, a B to the next 35%, and so on). In such cases, grades are inferred from the relative position rather than absolute performance, typically to prevent grade inflation and maintain consistency between lecturers.

Including both methods allows us to examine whether GPT-4’s performance varies depending on the nature of the grading task. Our aim is not solely to replicate prevailing human grading practices, but to explore how AI systems behave under distinct yet educationally relevant assessment frameworks. In particular, AI systems like GPT-4 may not follow the same performance patterns as human graders. Comparing the ranking task to the scoring task helps us investigate how different formats shape AI behavior and supports broader insights into the design of AI-assisted grading systems. For instance, it enables us to assess whether GPT-4 might benefit from access to comparative benchmarks when evaluating student responses.

Our quantitative approach compares the IRR of a team of three trained, independent human examiners with that of teams in which GPT-4 replaces one human examiner. IRR thereby measures the consistency in ranking or awarding points between raters for the same answers. A high IRR implies that ratings are not significantly influenced by personal biases, errors, or subjective interpretations, and thus more accurately capture the underlying truth. For example, a team of three human examiners achieving an IRR of 0.85 suggests reasonable convergence towards the ground truth. The key to our analysis, however, is not the absolute level of IRR, but how it changes when we replace one human examiner with GPT. If the IRR drops to 0.80, this would indicate GPT’s inferiority. Conversely, an increase to 0.90 would imply that GPT provides evaluations that are closer to the ground truth than those of humans. Our novel design thus allows us to abandon simplifying assumptions on human examiners grading without errors. Instead, we can compare examiners with varying degrees of fallibility.

Additionally, we investigate whether GPT has a self-serving bias, favoring its own answers over those written by humans. Previous studies have raised the question of whether GPT is prone to provide eloquent yet factually incorrect content^27,28^. Such a tendency may concur with a propensity to regard persuasive but baseless assertions as true in open-text answers. In particular, such a bias might show if GPT is more inclined than human examiners to fall for ‘eloquent bullshit’, to prefer form over substance. In an educational context, this would be problematic as it disadvantages students who express their knowledge differently. Moreover, examiners and students both using GPT-like models for generating and grading answers could initiate a slippery slope. Examiners might preferentially score AI-generated answers, thereby encouraging students to increasingly depend on these technologies for producing answers. This undermines the educational objective of fostering independent thinking and learning. Similarly, teachers might be misled by observing improvements in scores generated by GPT-like models, mistakenly interpreting them as genuine educational advancements. Ultimately, it would not be generative AI producing output similar to that of humans; it would be humans aligning their output to that of generative AI.

To capture a potential confound to GPT’s grading capabilities, we also examine whether GPT-4 has a bias towards longer text answers. The concern is that GPT is trained on a diverse internet corpus where detailed, comprehensive responses are often associated with higher quality information. It may then equate length with thoroughness or credibility, potentially favoring longer and more articulate responses even if they are not more accurate. We thus address the following two research gaps:

- **RQ1** How well does GPT-4 perform in ranking and assessing student answers as compared to fallible human examiners?
- **RQ2** Is GPT-4 biased by favoring GPT-generated answers or longer answers?

Foreshadowing our answers to these questions, we find that GPT-4 performs on par with human examiners in ranking student answers. GPT-4 does not show a more pronounced bias for AI-generated or lengthy answers than its human counterparts. We show that these findings are robust to tilting the competition to one side’s advantage, by using inferior or advanced prompting strategies. Our results are more attenuated for the point assessment, where we find that GPT-4 is more tolerant in assigning points and shows an excess bias on lengthy answers. This allows us to draw cautious but far-reaching implications for the use of GPT-4 and similar AI models in the assessment and grading of student examinations in higher education.

## Material and methods

### Data and procedures

We address our research questions based on student answers to open questions in an undergraduate course in macroeconomics collected during exercise classes between April and July 2022 using classEx^29^. classEx is an online tool for interactive classroom experiments and exercises that enables anonymous participation using personal mobile devices (smartphone, tablet, laptop). Anonymity is guaranteed by programming protocols (e.g. data encryption, use of pseudo-IDs). Over the course of one semester, students answered six open questions aligned with the progress of the class. Participation was voluntary, with students given 5–10 minutes to write a text answer during the exercise class. Answers were written in German. Thereafter, 2–3 student answers were randomly drawn and shown on the lecturer screen. The lecturer discussed them openly based on a sample solution and chose the best displayed answer to receive a prize of EUR 10.00 in a transparent and fair way. Hence, students were incentivized to write good answers. Apart from the monetary incentive, there were no external incentives in the form of credit or points awarded for a final exam. Importantly, we cannot link the answers to individual students. Students were orally informed beforehand that their input would be stored anonymously and could be used for research purposes, to which they consented by participating in the exercise.

In total, 517 student answers were collected. The number of answers per question varied substantially, ranging from 25 to 126. Answers contain on average about 275 characters with considerable variation across students and questions. They also differ in format, style and quality. An example of a question, sample solution and five student answers is given in Table 1 (translated to English). It illustrates the complexity of the task. Assigning points and comparing the quality of answers requires substantial knowledge of the underlying growth model, an identification of shortcomings and the steps of reasoning that students must undertake. For all six questions and sample solutions refer to 1 of the Supplementary Material.

**Table 1:** Example of open question, sample solution and 5 student answers.

*Question*	
How does the high population growth rate in Africa influence the level of the domestic product and its growth? Justify your answer with the help of the growth model!	
*Sample solution*	
A high population growth rate, as it is the case in many African countries, implies that the total capital stock is distributed among an increasing number of people. A low per capita capital stock results in a low level of per capita domestic product. However, the domestic product rises considerably due to the high population growth rate.	
*Student answers*	
The high population growth rate in Africa influences the level of domestic product and its growth. This is confirmed by the correlation between birth rate and growth in per capita income. Causality can also run the other way round.	
The higher the population growth rate, the lower the domestic product in Africa as the necessary per capita investments are higher.	
A high population growth rate means, conversely, that the capital stock must now be divided among more people. A smaller per capita capital stock means that productivity falls and with it GDP. Therefore, the aim in an economy should be: dN<dk.	
Growth rate n depends on the depreciation rate delta and the per capita capital stock k. If n increases, the per capita capital stock k automatically increases. A new steady state is formed in which the necessary investments are> than the actual investments.	
The high population growth rate in Africa has a negative impact on domestic product growth. As the necessary investments increase more strongly due to rapid population growth, the actual investments must increase all the more in order to achieve a steady state (necessary investments = actual investments).	

**Figure 1:**
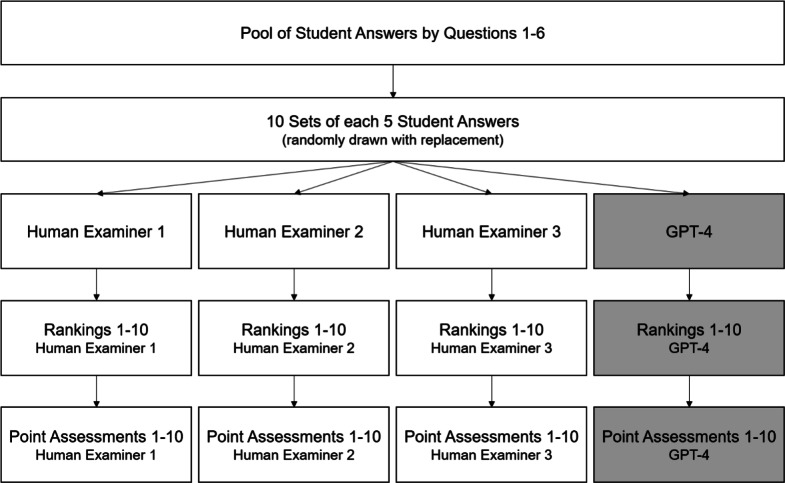
Procedure—ranking and point assessment of answers by human examiners and GPT-4.

To compare the performance of three human examiners and GPT-4 in assessing student answers, we proceed as illustrated in Fig. 1: For each of the six questions, we randomly draw 10 sets of five student answers each (with replacement). The example provided in Table 1 corresponds to one such set. We restrict the number of answers per set to five answers to keep comparisons cognitively manageable. Examiners have to relate all answers of a set to each other (as well as to a sample solution), such that each additional answer in a set increases the cognitive load over-proportionately. We observed upfront that larger sets become too demanding to human examiners, given the level of complexity of the student answers. For example, ranking five answers requires 10 pairwise comparisons (15 taking into account the sample solution) while for 8 this would increase to 28 (36 including the sample solution). Our choice of 10 sets per question ensures limited repeated selections of answers. At the same time, choosing the same number of sets per question independent of the number of collected answers entails that each question is given equal importance. Our full dataset comprises 300 observations, corresponding to 10 sets of five student answers for each of the six questions. To address RQ2 on the self-serving bias, we include further answers generated by GPT-3.5-turbo-instruct between March and May 2023. To achieve some variation in the quality and content of the GPT-generated answers, we varied the accuracy and relevance of the content provided to GPT. We set the temperature to zero and all other hyperparameters to the default values. The GPT-generated answers are randomly included in each set of student answers. They take the place of a student answer such that all sets consist of 5 answers each. In total, 39 of the 60 sets have one student answer replaced by a GPT-generated answer. For full access to the prompts and procedures of the GPT-generated answers see Section S2 in the Supplementary Material. Human examiners and GPT-4 were left ignorant about some of the answers being GPT-generated. Instead, they were led to believe that all answers were student answers. We debriefed human examiners about this fact upon completion of this study.

We provide the 10 sets along the open question, the respective sample solution and instructions to three human examiners and GPT-4. Their first task is to rank the five student answers in each set by quality based on the sample solution – from rank 1 (best answer of the set) to rank 5 (worst answer). Each rank can only be assigned once. The examiners work individually. Hence, for each set we receive three rankings from our human examiners and one from GPT-4. Their second task is to award points for each of the five answers in a set based on the sample solution. In this task, the provided sample solution is amended by further information about the contents for which points are to be awarded – similar to solution templates used to grade exams. The maximum score differs depending on the question between 3 and 5. The sets are the same for both tasks and all four examiners. We implement the two tasks as two independent alternative processes of examination since they may well yield different results. In particular, the ranking of answers is independent of the level of tolerance of examiners and enforces a constant distribution across the scale for grading. Contrary to this, points can be given with higher or lower tolerance and exhibit differences in variance and skewness across examiners. Task 1, the ranking, is always performed before Task 2, the point assessment. The human examiners performed both tasks between March and June 2023. GPT-4 performed the ranking task in June 2023 and the point assessment in August 2023.

Our human examiners are three student assistants employed at the Chair of Economic Theory who have successfully passed the course in macroeconomics with grades high above average. They can be considered trained experts in this field and are qualified to correct undergraduate exams in macroeconomics. The student assistants partook in the study not as regular experimental subjects but as employees of University of Passau. They were informed and consented that their work product would be used for the purposes of this study. We access GPT-4^11^ via chat completion API provided by OpenAI^30^. The instructions provided to the human examiners are kept as similar as possible to the prompt provided to GPT-4 (see Section S3 of the Supplementary Material). This helps us level the playing field between the human examiners and GPT-4. We thus avoid any bias caused by different instructions or prompt overfitting on the study. Nevertheless, we evaluate different prompting strategies for purposes of robustness in the Section “Robustness checks and extensions”.

Our entire study protocol has been reviewed and approved by the Research Ethics Committee of the University of Passau. We confirm that our study protocol adheres to all pertinent guidelines and regulations.

### Experimental design and hypotheses

**Figure 2:**
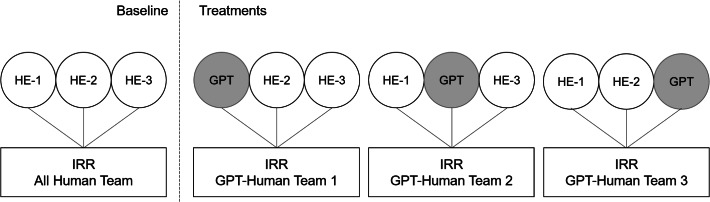
Experimental design—baseline and treatment scenarios.

To address the question whether GPT-4 can substitute human examiners (RQ1), we employ a simple yet novel experimental design as illustrated in Fig. 2: We consider the ranking and point assessment by our three human examiners (HE) as the baseline scenario. We compare this baseline scenario with three treatment scenarios. In each, one of the human examiners is replaced by GPT-4. These scenarios are classified as GPT-Human Teams 1, 2, and 3. For comparison between baseline and treatment scenarios we use the IRR, again determined by the consistency in ranking or point assessment between raters (GPT-4 and the three human examiners) for the same answers, among the three examiners in each team. Given that the true rankings and scores have not been disclosed in the Web our approach is also not prone to data-poisoning^31^.

This metric allows us to evaluate how consistent the members of each team are at ranking and assigning points. A high IRR implies that ratings are not significantly influenced by personal biases, errors, or subjective interpretations. Ratings then more accurately capture the underlying reality. The metric thus offers a comparative measure of performance between All Human and GPT-Human Teams. Our novel design allows us to abandon simplifying assumptions on human examiners grading without errors. Instead, we can compare between examiners that have varying degrees of fallibility. A decrease in IRR when GPT-4 is introduced as the third examiner indicates that GPT-4’s judgment capability is less aligned with the remaining two human examiners than the replaced human examiner. GPT-4 would then perform worse than humans. In contrast, an increase in IRR demonstrates that GPT-4’s assessments perform better than those by humans.

We investigate potential differences in the IRRs between baseline and treatment scenarios separately for the ranking and the point assessment task. This allows us to address RQ1 in its two relevant dimensions: Firstly, it explores the capability of GPT-4 to measure the relative quality of student answers as necessary to determine grades, in particular when applying grade curves (ranking). Secondly, we examine GPT’s ability to distinguish between correct and incorrect content to award scores (point assessment). We expect the treatment effects to go in the same direction for both tasks since the challenges examiners face are similar. Ranking and point assessing answers to open questions requires an understanding of intricate reasoning, subtle nuances, and creative thinking that leave some room for inventive answers beyond the sample solution. Recent studies suggest that these challenges may be greater for GPT than for human examiners since generative-AI models have shown to lack reasoning competencies^11,22,28,32^. As GPT-4 reflects aggregate opinions present on the internet, we further asked GPT-4 itself to form expectations on its performance as compared to human examiners in our specific tasks. In line with the previous findings, GPT-4 replied: “In both tasks – ranking answers and awarding points – human examiners will likely outperform GPT-4 because they can better understand subtleties, context, and originality in student answers. While GPT-4 can handle structured tasks more effectively, its understanding of complex reasoning and unconventional solutions is limited.” Similarly, we hypothesize that GPT-4, solely relying on the patterns it observed in its training data, cannot achieve the accuracy and comprehensiveness of the human mind:

- **H1** [IRR]: The All Human Team achieves a higher IRR than teams that include GPT-4 (GPT-Human Teams).

Further, we are interested in whether there is a self-serving bias in the rankings and point assessments of GPT-4 (RQ2). If such a self-serving bias exists, GPT would rank and point assess the GPT-generated answers systematically better than its human counterparts. This would pose a major problem for the use of GPT as an examiner since students who have their answers written by GPT-like models would receive better grades. There are two reasons why this bias might occur: First, GPT might be inclined to judge GPT-generated answers more favorably due to the linguistic proximity and correspondence with the training data. Second, and relatedly, GPT answers tend to be more sophisticated in terms of language than student answers. Yet Rudolph et al.^28^ emphasize that when it comes to critical, analytical thinking, AI lacks competence. If GPT prefers eloquent but less competent answers, this might likewise reflect in this bias. We hence hypothesize:

- **H2** [Self-serving bias]: GPT-4 judges GPT-generated answers more favorably than human examiners do.

Moreover, GPT-4 might be biased towards longer answers^33,34^. First, this might be due to longer answers being more eloquent. Second, whereas the human mind recognizes repetitions of content and does not value them as additional input, GPT-4 might not be able to apply such critical assessment, confusing length with accuracy. Even if humans are not immune to such an influence, it might be more pronounced for GPT-4. Thus, we hypothesize:

- **H3** [Length bias]: GPT-4 judges long answers more favorably than human examiners do.

### Prompting strategy

In our prompt, we follow the zero-shot learning technique^3^, where we do not provide any example to the model as demonstration to guide its response. This levels the playing field between human examiners and GPT-4, enabling a one-to-one comparison without granting GPT-4 additional advantages^3^. Apart from some technical details, the prompt is thus entirely based on the instructions provided to our human examiners. We later abandon this setting to explore alternative approaches: First, a single-answer variant for point assessment in which GPT evaluates one answer at a time rather than sets of five, and subsequently, more advanced prompting techniques aimed at improving performance compared to zero-shot learning. Specifically, we use a variant of chain-of-thought prompting^35^, where we guide the model to generate reasoning behind its response.

We access GPT-4 via chat completion API provided by OpenAI^30^. The temperature is set to zero to approximate a deterministic behavior of GPT-4. GPT-4 and other large language models are inherently non-deterministic, yet a temperature of zero facilitates increased replicability. Chat completion API also provides a placeholder for system messages to act as a guide to the overall generation of the model. We have kept the system message empty to avoid any external bias on the model and to instruct human examiners and GPT-4 as closely as possible. Furthermore, each set of student answers was prompted independently to ensure that context from previous interactions does not influence subsequent prompts. All the remaining hyperparameters are kept unchanged as provided by the API. Our original prompts are in German. We refer to Section S4 of the Supplementary Material for the original prompts and their translations into English.

## Results

### IRR in ranking and point assessments

**Figure 3:**
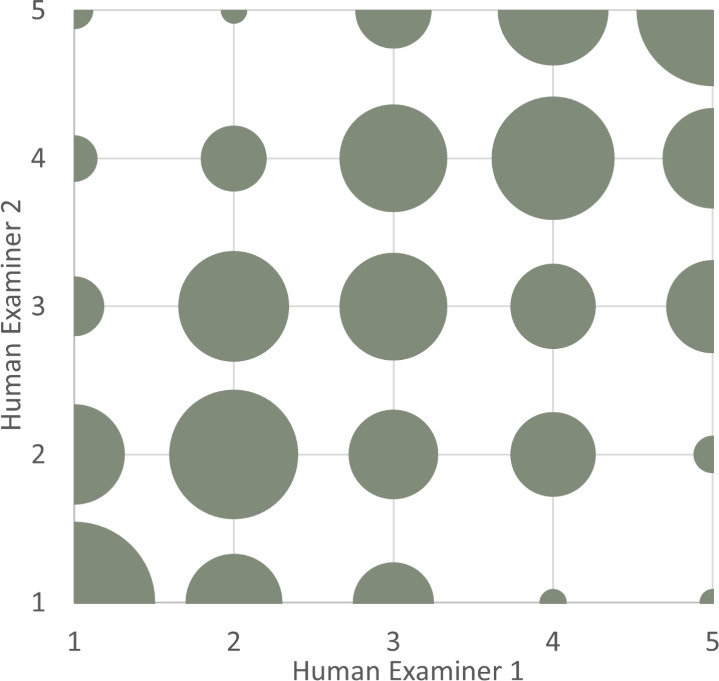
Bubble chart of ranks assigned by human examiners 1 and 2.

We test Hypothesis 1 – whether IRR drops when replacing a human examiner with GPT-4 – separately for our two tasks as different statistics are to be applied. For the ranking task, we use Quadratic Weighted Kappa (QWK) for pairwise comparison and Kendall’s W to quantify agreement between multiple raters. They are the preferred metric for measuring IRR when data is ordinal. To illustrate, Fig. 3 plots the agreement between two human examiners using a bubble chart. The 300 ranks assigned by human examiner 1 are on the x-axis and those assigned by human examiner 2 are on the y-axis. The size of the bubble indicates the frequency of the observations. The bubbles are visibly largest on the diagonal from bottom left to top right, diminishing in size as they diverge from this central axis. This shows considerable agreement between the examiners, but also the inevitable disagreement between well-trained humans. This figure corresponds to what is commonly referred to as a confusion matrix, which shows correspondence on the diagonal and deviations on points being off the diagonal. For sake of parsimony, we only report the confusion matrix for one pair. We obtain similar bubble charts for the remaining pairs.

To calculate the pairwise IRR between the two human examiners depicted in Fig. 3, we use QWK. Values can range from -1 to 1. A value of 1 indicates complete agreement, all the bubbles lie on the diagonal from bottom left to top right, while a value of -1 indicates complete disagreement, all the bubbles lie on the second diagonal from top left to bottom right. For this pair, we obtain a value of 0.648. We conduct the same analysis across all three human pairs as well as the GPT-human pairs and report results in Table 2. We get a value of 0.742 for human pair 1|3 and 0.670 for human pair 2|3. The average across the three human pairs corresponds to 0.687. Replacing a human examiner with GPT-4, we get pairwise values of 0.700 for IRR of GPT-4 with human examiner 1, 0.688 with human examiner 2 and 0.685 with human examiner 3. The average corresponds to 0.691 for these three pairs. It does not significantly differ from the average across the three human pairs (permutation test, $p=0.435$, see Section S6 of the Supplementary Material for details). The same holds for all pairwise comparisons between human and GPT-human pairs and is thus a robust finding (see Table 2). This is first evidence that GPT-4 performs equally well in ranking.

**Table 2:** Pairwise comparison results—ranking.

Pairwise comparison	Pair 1 (QWK)	Pair 2 (QWK)	Permutation test
1|2 vs. 1|GPT	0.648	0.700	p = 0.143
1|2 vs. 2|GPT	0.648	0.688	p = 0.210
1|3 vs. 1|GPT	0.742	0.700	p = 0.170
1|3 vs. 3|GPT	0.742	0.685	p = 0.110
2|3 vs. 2|GPT	0.670	0.688	p = 0.350
2|3 vs. 3|GPT	0.670	0.685	p = 0.377
All-Human vs. GPT-Human	0.687	0.691	p = 0.435

**Figure 4:**
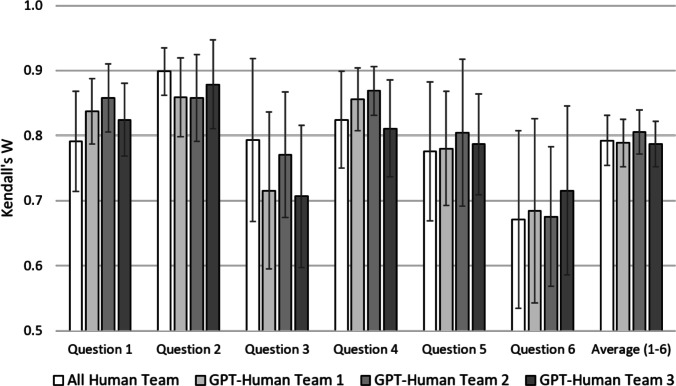
Results for the ranking task by question with 95 percent confidence intervals.

To quantify the agreement between *n* raters, where $$n\ge 2$$, we use Kendall’s W. It measures agreement on a range from 0 to 1. A score of 1 indicates complete agreement between the raters, while a score of 0 indicates no agreement. We calculate Kendall’s W separately for the All Human and the three GPT-Human Teams and then compare the outcomes. If Kendall’s W is significantly lower in the GPT-Human Teams, we find evidence in favor of Hypothesis 1. We illustrate our results in Fig. 4. As shown on the right, the overall average IRR is 0.792 for the All Human Team (marked by a white bar) and either stays at that level for the GPT-Human Teams or even increases to 0.806 for the GPT-Human Team 2, marked by the darker bars (for details and summary statistics, see Section S5 in the Supplementary Material). The overlapping confidence intervals indicate that there are no significant differences. We corroborate this finding by help of a Mann-Whitney U test. For this, we determine Kendall’s W separately for each of the 60 sets per team. Then, we test for equality of the average Kendall’s W between the All Human Team and each of the three GPT-Human Teams. The test results show no significant differences between the All Human Team and GPT-Human Teams 1 (*Z*=0.349, $$p=0.727)$$, 2 (*Z*=-0.293 $$p=0.770)$$, and 3 (*Z*=0.620, $$p=0.535)$$. Also for the six questions separately, each referring to 50 observations, the overlapping confidence intervals indicate no significant differences between the teams. Overall, Kendall’s W is higher in Question 2 and lower in Question 6, which could correspond to the quality of the students’ answers or the examiners’ ability to identify their respective quality. Both are likely to vary from question to question. However, this variation affects the All Human and the GPT-Human Teams equally. There is no question for which the GPT-Human Teams perform significantly worse.

**Figure 5:**
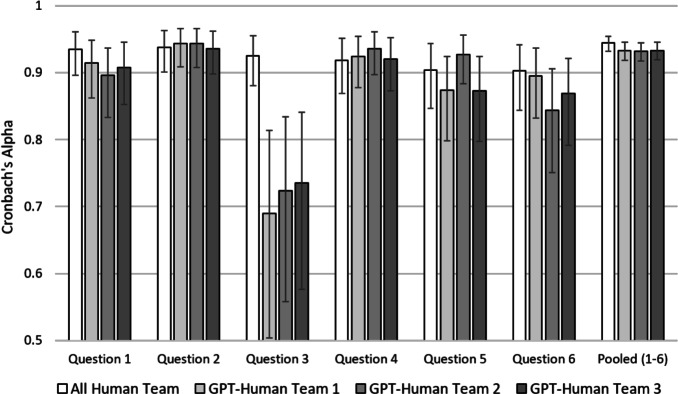
Results for the point assessment task by question with 95 percent confidence intervals.

The IRR for point assessments requires a slightly different approach. While ranks reveal ordinal information, point assessments are cardinal, meaning that a difference between 0 and 1 is equal to a difference between 1 and 2. The IRRs of our All Human Team and GPT-Human Teams should then be measured using Cronbach’s Alpha. Figure 5 illustrates our results for the point assessment. As can be observed, we now find a slight drop in the IRR for all GPT-Human Teams pooling all questions, with values of 0.933, 0.932 and 0.933 compared to the All Human Team, with a value of 0.944. This difference is however not statistically significant ($\chi ^2 = 1.96$, $p = 0.580$)^36^. Looking at effect heterogeneity across Questions 1–6, we detect drops in IRR between the All Human Team and all three GPT-Human Teams for Questions 1, 3, and 6. For Question 3, it is most pronounced in size and statistically significant ($\chi ^2 = 18.62$, $p < 0.001$)^36^. Nonetheless, there is no reason to treat Question 3 as an outlier. We qualitatively assess all questions on the required level of competence. We find that they are all closely aligned with the content of the course in macroeconomics and observe no noteworthy qualitative differences between them. Our results hence raise doubts about the capacity of GPT-4 to replace humans examiners when it comes to point assessments.

To corroborate this finding, we also look at correlations for the point assessment, using simple Pearson correlation coefficients. As shown in Table 3, we find values of 0.861 for pair 1|2, 0.856 for 1|3 and 0.836 for 2|3. The joint correlation among the three human examiners amounts to 0.847. The pairwise correlations between GPT-4 and the three human examiners amount to 0.795 with human examiner 1, 0.818 with human examiner 2 and 0.827 with human examiner 3. The joint correlation of GPT with the three human examiners is 0.812. There is thus again a noteworthy drop in reliability for GPT-4. The correlation between human examiner 1 and GPT-4 is significantly below that of human examiner 1 with human examiner 2 (permutation test, $p=0.028$, see Section S6 of the Supplementary Material for details on the determination of a joint correlation and all permutation tests) and 3 (permutation test, $p=0.032$). Also the correlation of human examiner 2 with GPT is somewhat lower than that of human examiner 2 with human examiner 1 (permutation test, $p=0.067$). It is only human examiner 3 who appears to be more closely aligned with GPT. We find no significant differences between the correlation of human examiner 3 with GPT and human examiner 3’s correlation with human examiner 1 (permutation test, $p=0.151$) or human examiner 2 (permutation test, $p=0.377$). The correlation among the three human examiners of 0.847 is significantly higher than the correlation of 0.812 between GPT-4 and the three human examiners (permutation test, $p=0.024$).

**Table 3:** Pairwise comparison results—point assessment with five answers to score.

Pairwise comparison	Pair 1 (Pearson)	Pair 2 (Pearson)	Permutation test
1|2 vs. 1|GPT	0.861	0.795	p = 0.028
1|2 vs. 2|GPT	0.861	0.818	p = 0.067
1|3 vs. 1|GPT	0.856	0.795	p = 0.032
1|3 vs. 3|GPT	0.856	0.827	p = 0.151
2|3 vs. 2|GPT	0.836	0.818	p = 0.253
2|3 vs. 3|GPT	0.836	0.827	p = 0.377
All-Human vs. GPT-Human	0.847	0.812	p=0.024

Why does GPT-4 perform well on a ranking task and less well on a point assessment task? A ranking task requires the comparison of five answers relative to each other. A higher rank for one means a lower rank for another. A point assessment task allows more points to be assigned to the sum of all five answers, permitting one to perform better without downgrading the other. A point assessment task thus gives examiners a degree of freedom in determining the level of tolerance. Some will be tolerant and show leniency in awarding points, while others will be stricter.

There are in fact major differences in tolerance between GPT-4 and the human examiners. Across all questions, the human examiners award on average 1.30 (standard deviation 1.27), 1.23 (1.30), and 1.35 (1.17) points, showing only small differences. GPT-4, on the other hand, stands out with an average of 1.81 (1.36). The mean does not significantly differ between the three humans subjects (permutation test, $p=0.285$ for pair 1|2, $p=0.296$ for 1|3 and $p=0.121$ for 2|3, see Section S6 of the Supplementary Material for more information), but GPT-4’s mean is significantly higher than that of any human examiner (permutation test, $p<0.001$ for any human). The instructions, potentially supported by prior experience with similar tasks, appear to have aligned the human examiners’ tolerance levels, whereas GPT-4’s broader, more generic training data may underlie its substantially greater tolerance. This tolerance may also reduce the IRR. For example, GPT-4 might be more tolerant than humans when judging the completeness of an answer while being similar in other dimensions, such as accuracy or the willingness to detect valid points anywhere in the sample solution. Such a selected tendency might then reduce the correlation with human assessments.

Can GPT-4’s performance in the point assessment task be improved by simplifying its evaluation process? In the initial setup, GPT-4 scored answers in sets of five, mirroring the procedure employed in the ranking task and given to the human examiners. We now modify this by having GPT-4 score each answer individually, which may reduce the complexity of the task and improve performance (for the prompt see Section S7 of the Supplementary Material). The results support this hypothesis. Reliability, measured by Cronbach’s Alpha, increases to 0.942 for GPT-Human Team 1, 0.941 for GPT-Human Team 2 and 0.944 for GPT-Human Team 3, now approaching the value of 0.944 for the All Human Team. In line with these findings, the pairwise comparisons also no longer differ significantly between the GPT-human and human-human pairs (see Table 4). This suggests that, unlike human examiners who can ignore non-relevant answers in a set, GPT-4’s performance is adversely affected by their presence.

**Table 4:** Pairwise comparison results—point assessment with single answer to score.

Pairwise comparison	Pair 1 (Pearson)	Pair 2 (Pearson)	Permutation test
1|2 vs. 1|GPT	0.861	0.829	p = 0.124
1|2 vs. 2|GPT	0.861	0.854	p = 0.394
1|3 vs. 1|GPT	0.856	0.829	p = 0.149
1|3 vs. 3|GPT	0.856	0.849	p = 0.372
2|3 vs. 2|GPT	0.836	0.854	p = 0.759
2|3 vs. 3|GPT	0.836	0.849	p = 0.682
All-Human vs. GPT-Human	0.847	0.842	p = 0.388

### Self-serving bias and length bias

Even if GPT-4 performs similarly to human examiners, there might still be reservations against using it broadly for examination tasks. Its ranks and points might show a problematic bias. GPT-4 could rate GPT-generated answers more favorably than humans would. Such a bias would imply a slippery slope with students using GPT to write answers and teachers simultaneously using GPT for grading, indicating an excessively favorable performance to both. A similar problem arises with lengthy answers, which might be indicative of ‘eloquent bullshit’ performing too well, inducing students to write lengthy answers with little content. While overall agreement with human examiners is relatively high, indicating that substantial discrepancies are unlikely, the performance metrics used may mask systematic divergences in specific categories such as in GPT-generated or lengthier answers. We therefore examine the potential for such biases more explicitly using an ordered probit regression:

1 $$\begin{aligned} & GPT_i=\sum _{j=1}^3{\beta _j Human_{ji}} + \gamma _1 GPTA_i +\gamma _2 Length_i + \sum _{k=1}^6 {\delta _k Question_{ki}} + \epsilon _i & \end{aligned}$$

where *GPT* and *Human* refer to ranks and points assigned by GPT and our three human examiners, respectively. The subindex *i* refers to our 300 observations stemming from the 10 sets of five answers for each of the six questions. The subindex *j* refers to one of three human examiners. $GPT_i$ is our dependent variable and we test how well it is explained by $Human_{ji}$ and potential biases. The first such bias is the dummy $GPTA_i$, which takes the value of 1 if a graded answer was written by GPT and 0 otherwise. $Length_i$ is the second potential bias, indicating the length of an answer in 10 characters. $Question_{ki}$ is also a dummy that takes the value 1 if the answer *i* refers to the question *k* and obtains the value 0 otherwise. We include this to account for unobserved heterogeneity between questions.

We report results in Table 5. The positive and large coefficients on human examiners 1, 2 and 3 corroborate our result that GPT ranks in line with our human examiners, while showing also a strong influence of the points assigned by human examiners on those awarded by GPT. This holds true for each of our three human examiners. Concerning the self-serving bias, our coefficient of interest is on the GPT Answer. If GPT has a bias towards preferring GPT-generated answers, we expect a negative coefficient for ranks — as low ranks are assigned to better answers - and a positive coefficient for points, as better answers achieve more points. Given that we control for the assessments by the human examiners, these coefficients indicate whether GPT deviates from the human examiners when assessing its own answers. Indeed, we find point estimates for the coefficients that follow this pattern. However, both for ranking (Column 1) and points (Columns 2 and 3), coefficients are very small and not significantly different from zero. We therefore see no evidence substantiating Hypothesis 2. We find that GPT does *not* judge GPT-generated answers more favorably than human examiners.

Regarding Hypothesis 3 and the question of whether GPT has a bias towards longer text answers, the coefficient on *Length* in Table 5 suggests that there is no bias towards preferring longer text answers when ranking answers (Column 1). While the coefficient is negative, it is very small in size and not significantly different from zero. When it comes to points, the picture looks somewhat different (Columns 2 and 3). Here, both for the five- and single-answer point assessment we indeed find that GPT gives longer answers more points than our three human examiners. This result holds accounting for Bonferroni correction. It suggests that GPT has a bias towards length when awarding points but not when ranking student answers. This evidence can be plausibly related to GPT’s excess tolerance. The three human examiners assign on average 0.94 points to short answers (those below and equal to the median of 277 characters) and 1.68 points to long answers (those above 277 characters). In contrast, GPT-4 assigns 1.33 points to short answers — 0.39 points more than humans — already showing excess tolerance (1.07 points, or 0.13 points more than humans in the single-answer scoring). The difference, and thus excess tolerance, is even larger for long answers: GPT-4 assigns 2.33 points, 0.65 points more than humans (1.97 points, or 0.29 more in the single-answer scoring). In line with previous work^33,34^, this pattern suggests that GPT’s tolerance is partly linked to its preference for length. Excess tolerance is not possible in rankings tasks, implying that a source of bias is removed on these tasks. This might then provide an explanation for the better performance of GPT in the ranking tasks.

**Table 5:** Ordered probit regression results on self-serving and length bias.

	(1)	(2)	(3)
	GPT-4 Rank	GPT-4 Points (Five Answers)	GPT-4 Points (Single Answer)
Human Examiner 1	0.36***	0.26**	0.37***
	(0.07)	(0.1)	(0.1)
Human Examiner 2	0.38***	0.53***	0.74***
	(0.06)	(0.1)	(0.1)
Human Examiner 3	0.28***	0.78***	0.77***
	(0.07)	(0.1)	(0.1)
GPT Answer	-0.11	0.17	0.27
	(0.2)	(0.2)	(0.2)
Length	-0.0055	0.032***	0.037***
	(0.008)	(0.008)	(0.009)
Question Dummies	Yes	Yes	Yes
Observations	300	300	300
Pseudo $R^2$	0.27	0.46	0.49

Standard errors in parentheses.

*$$p<0.10$$, **$$p<0.05$$, ***$$p<0.01$$.

To confirm potential heterogeneity in the length bias, we conduct a split-sample regression, running Equation (1) separately for answers at or below the median length of 277 characters, and for those above. The bias is most pronounced for longer answers and becomes statistically insignificant — even at the 10% level — for shorter answers (see Section S8 of the Supplementary Materials). We also test targeted prompting strategies to mitigate this bias, as reported in Section S8. One approach adds explicit instructions to ignore length. The other approach employs a two-step reasoning prompt in which GPT-4 first checks, for each point in the sample solution, whether it is present in the student’s answer or not, and then counts the number of confirmed points to determine the score. This process mirrors how human graders typically use rubrics and prevents duplications in a student’s answer from inflating its score. Still, the bias persists in both approaches, remaining virtually unchanged with the explicit instructions to ignore length but halving in size with the two-step prompt.

### Robustness checks and extensions

Our focus on open-text answers in macroeconomics is quite specific. Whether our results are robust across further domains, disciplines, lengths of answers, or variations in style and content will have to be seen and is outside the scope of our study. Using our current dataset we can however address another challenging question: To what extent do our findings depend on the current version of GPT-4 and variations in the design of the prompt? We also ask whether we can boost the performance of GPT-4 by using an advanced prompt strategy. Examiners who employ GPT-4 as an assistant are likely to use different prompts and approach GPT-4 at different points in time. Would such variations matter to our results?

We employ the following checks to answer these questions: We re-run our original prompt version $P_{v1}$ with a later version of the model (approximately nine months apart) to ensure that our findings are robust to model drift^37,38^, for example induced by real-world data or user behavior evolving over time and exerting an impact on GPT. Additionally, we investigate the effect of four design variations chosen based on previous literature to determine how robust our prompt is to certain manipulations. Our first variation refers to the structure of the prompt^39^. We modify its structure without altering the content, employing four different orders ($P_{v2}$ – $P_{v5}$). The second refers to linguistic variability^40^. $P_{v6.1}$ ($P_{6.2}$) randomly introduces a considerable (limited) number of heavy (minor) spelling errors to the text. $P_{v7}$ alters the tone of the prompt introducing substantial ambiguity. The third variation refers to roles^41^. We alter the role of GPT-4 from student assistant to $P_{v8}$ professor, $P_{v9}$ expert student assistant, $P_{v10}$ non-expert student assistant, and $P_{v11}$ no role. In the last variation $P_{v12}$, we introduce (zero-shot) chain-of-thought (CoT) prompting^42^, where we guide the model to generate reasoning behind the ranks and points given. It has been shown to achieve superior performance in reasoning tasks^35^. Refer to Section S9 of the Supplementary Material for all prompts.

**Figure 6:**
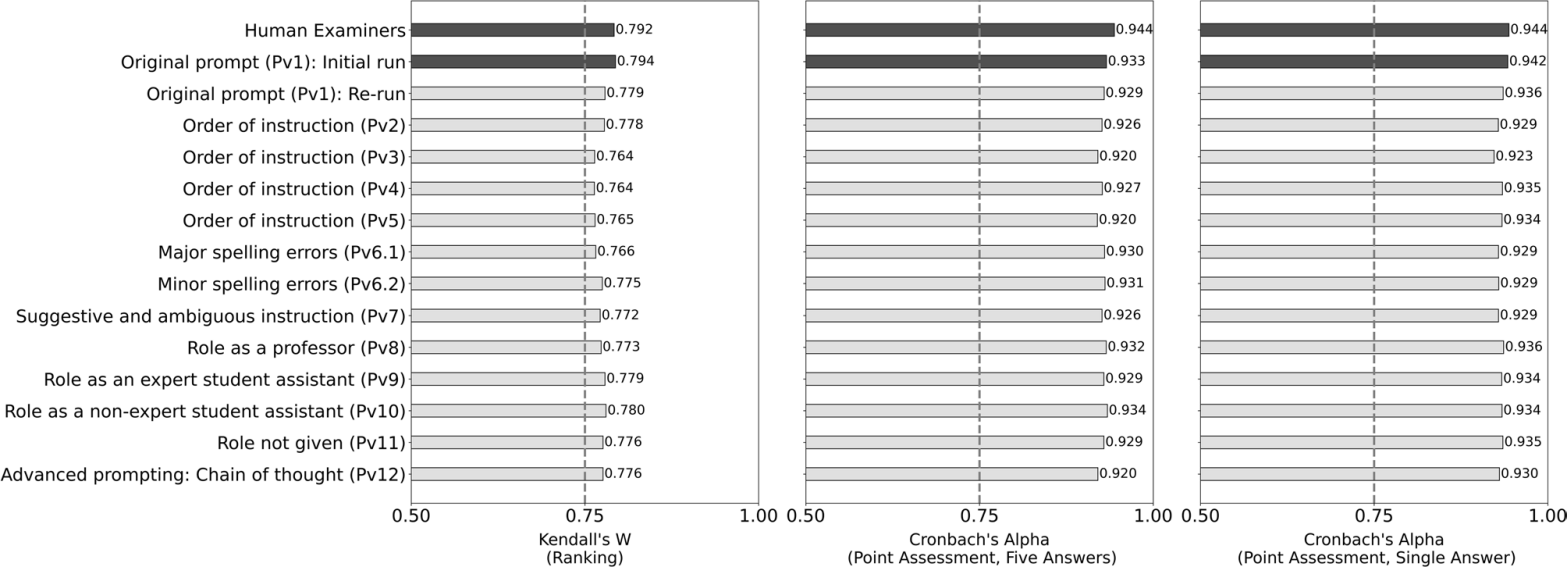
Robustness of GPT-4 across varied run times and prompts in ranking and point assessment.

Figure 6 shows that using GPT-4 as an examiner generates findings that are persistently close to those of human examiners. For reference, the bars in the first line depict our baseline All-Human team for the ranking and point assessment, respectively. The bars in the second line correspond to the average of the GPT-Human teams. The following bars refer to the different robustness checks. We aggregate results for the three GPT-Human teams. For disaggregated results and error measures see Section S10 in the Supplementary Material. Kendall’s W and Cronbach’s Alpha drop slightly but not substantially in the re-runs of the original prompt. Similarly, we observe that variations in the prompt design appear to play a subordinate role: Changes to the structure of the prompt (as employed in $P_{v2}$ – $P_{v5}$) decrease IRRs in all treatments but only slightly. Likewise, spelling errors ($P_{v6.1}$ and $P_{v6.2}$) as well as suggestive and ambiguous instructions ($P_{v7}$) in the prompt have a dampening yet negligible effect. Variations in the role assigned and not assigned to GPT-4 as indicated by $P_{v8}$ – $P_{11}$ do not considerably alter our results. Finally, we find that introducing an advanced prompting strategy, $P_{v12}$ does not bring any improvement over the original prompt $P_{v1}$.

## Discussion and conclusions

Undoubtedly, GPT-4 poses major challenges to the education system, but it also offers big opportunities. The latter could lie in its potential to reduce teachers’ workload – for example, by letting GPT-4 grade and assess student answers. Two essential requirements to grade and assess student answers are the capability of GPT-4 to measure the relative quality of student answers as necessary to determine grades (ranking) as well as to distinguish between correct and incorrect content to award scores (point assessment). This study investigates whether GPT-4 can actually replace human examiners in ranking and point-assessing student answers to open questions.

Particularly for the ranking tasks, our results give overworked teachers cause for optimism: GPT-4 can recognize differences in the quality of student answers as reliably as trained human examiners. We do not detect any bias towards GPT-generated answers or the length of answers in the ranking assessment of GPT-4. Hence we can rule out that GPT-4 is inclined to fall for eloquent bullshit or linguistic proximity to its own model. Concerns that students using AI for their answers may gain an advantage in their teacher’s assessment seem unfounded.

In the point assessment task, GPT-4’s performance is promising but raises further questions future research needs to address: When grading sets of five answers, there is a slight decrease in IRR from the All Human Team to the GPT-Human Teams. This may be due to a greater tolerance in the point assessments of GPT-4. Moreover, the length of answers positively affects the number of points awarded by GPT-4, indicating that a conflation of length with accuracy cannot be ruled out. Converting the task from batch grading to single-answer scoring increases the IRR, bringing overall performance to the same level as that of the All Human Team. This suggests that minimizing complexity is important for GPT to reliably award points; rather than multiple student answers at once, GPT should be given each answer individually when being utilized for point assessment. However, also in this simplified setting, the impact of answer length persists and remains significant. This length bias is seemingly robust — neither specific instructions to ignore length nor an alternative prompting strategy designed to limit excess tolerance eliminates it. Following our median split analysis, an approach of mitigating it could be to strictly limit the maximum number of characters students can write, as the bias is particularly pronounced for longer student answers.

Given its more reliable performance on the ranking task, a potential way forward would be to assist GPT in the point assessment by supplying predetermined answers that span the range of possibles grades. For example, answers that earn 1 point, 2 points, 3 points and 4 points may be compared to a newly submitted student answer. The ranking by GPT might then allow for an improved point assessment. Future research should focus on refining this method and testing how best to assist GPT with such predefined answers. Another conclusion would be that GPT performs better when grade curves are applied. Thus, there are specific forms of examinations where GPT might be able to assist in grading tasks.

Our study has a few further caveats to consider when drawing implications: The open-ended questions asked are not ambiguous – there is only one correct solution. Therefore, the external validity of our findings is limited to similar types of questions. Potentially, GPT-4 is not as competent in differentiating between multiple correct answers. Future research should thus examine whether the results hold for more complex questions that allow for more than one correct argumentation. Similarly, our rankings are based on sets of 5 answers each. It remains an open question of how GPT fares ranking larger sets of answers. Cognitive limitations that might restrict humans’ ability to efficiently rank larger sets of answers may not apply to GPT. Furthermore, our results relate to the field of macroeconomics. The question of how well GPT can rank and point assess student answers in other disciplines also remains a matter for future research. Another regard in which the external validity might be limited is the language. We conducted our study in German. GPT-4’s accuracy in German is at a quite similar level as in English^11^, yet its performance in ranking and point assessing student answers might be different in languages it is less proficient in. Also in this regard, further testing should be conducted. Relatedly, our results are limited to the use of GPT-4. We cannot draw any inferences about the use of other large language models in assessing open-text answers.

Eventually, there are some ethical and legal considerations on the part of the teachers to take into account: We do not recommend that teachers pawn off grading exams and essays to GPT-4 without any control or supervision. Ultimately, grading and assessing students’ work lies in the responsibility of the teacher. There is currently no legal basis to transfer this responsibility to AI. An AI cannot be held responsible in case of misgrading. This falls under the teacher’s purview. We thus only cautiously recommend using the AI’s grading and assessment capabilities as a replacement to a teacher’s assessment in settings where grades carry little importance. When it comes to a complementary role to teacher’s assessment, GPT-4’s evaluation can serve as an additional layer of oversight, allowing for broader use without the same limitations. For example, comparing a teacher’s point assessments with the ranks that GPT-4 would assign is likely to provide useful feedback on the quality of grading.

## Supplementary Information

Supplementary Information.

## Data Availability

The datasets generated during and/or analyzed during the current study are available in Zenodo repository, https://doi.org/10.5281/zenodo.11085379.

## Code availability

A link to a replication package that contains the data and the analysis code is also available in Zenodo repository, https://doi.org/10.5281/zenodo.11085379.
