# Supplementary material for: GPT-4 shows comparable performance to human examiners in ranking open-text answers
Source: Sci Rep. 2025 Oct 8;15:35045. doi: 10.1038/s41598-025-21572-8 (PMC12508078; doi:10.1038/s41598-025-21572-8)
Supplement: Supplementary file 1 — Supplementary Information. [file 41598_2025_21572_MOESM1_ESM.pdf]

GPT-4 Shows Comparable Performance to Human Examiners in Ranking Open-Text Answers

Abdullah Al Zubaer, Michael Granitzer, Stephan Geschwind, Johann Graf Lambsdorff, and Deborah Voss

S1 Questions and Sample Solutions

In the following we provide the six questions and respective sample solutions both in the original German version and translated into English:

Question 1

Question: Inflation hat viele negative volkswirtschaftliche Konsequenzen, aber auch positive Auswirkungen. Bitte erklären Sie, wie Inflation die Flexibilität der Löhne positiv beeinflusst.

Inflation has many negative economic consequences, but also positive effects. Please explain how inflation has a positive impact on wage flexibility.

Sample Solution: Löhne sollten mit erhöhter Produktivität eines Unternehmens steigen. Sinkt in einem Unternehmen die Produktivität, beispielsweise in sterbenden Branchen, so sollten in diesem die Löhne dementsprechend sinken. Eine solche Lohnsenkung wird jedoch von ArbeitnehmerInnen als unfair wahrgenommen und kann zu einer verringerten Arbeitsmoral und möglicherweise sogar zu Streiks führen. Bei Vorhandensein von Inflation kann eine Lohnsenkung dadurch erfolgen, dass Löhne nominal konstant bleiben und durch die Preissteigerung real entwertet werden. Dies wird oft als weniger unfair empfunden. So kann Inflation Unternehmen mehr Flexibilität geben, um eine unvermeidliche Lohnsenkung durchzuführen.

Wages should rise as a company's productivity increases. If productivity falls in a company, for example in dying industries, wages should fall accordingly. However, such a wage reduction is perceived as unfair by employees and can lead to reduced morale and possibly even strikes. In the presence of inflation, wages can be reduced by keeping nominal wages constant and having them be devalued in real terms by not adjusting them to price increases. This is often perceived as less unfair. Inflation can thus give companies more flexibility to implement an unavoidable wage reduction.

Question 2

Question: Was versteht man unter der Makroökonomik als engineering und wie sieht diese menschliches Verhalten und staatliche Eingriffe?

What is meant by macroeconomics as engineering and how does it view human behavior and government intervention?

Sample Solution: Die Makroökonomik als engineering hat ihren Ausgangspunkt in der Beobachtung von Interaktionen am Markt. Aus diesen Beobachtungen wird eine Theorie abgeleitet, die folgende Annahmen zugrunde legt: Marktteilnehmer handeln nicht immer rational, weisen oftmals ein Herdenverhalten auf, und sind in ihren Entscheidungen von Stimmung und Gewohnheit beeinflusst. Aus diesen Gründen findet der Markt oft nicht von selbst zum Gleichgewicht, weshalb Eingriffe des Staates aus der Sichtweise des engineerings notwendig und wünschenswert sein können.

Macroeconomics as engineering is based on the observation of interactions on the market. From these observations, a theory is derived that is based on the following assumptions: Market participants do not always act rationally, often exhibit herd behavior, and are influenced in their decisions by mood and habit. For these reasons, the market often fails to reach equilibrium on its own, which is why state intervention may be necessary and desirable from an engineering perspective.

Question 3

Question: Wie beeinflusst die hohe Bevölkerungswachstumsrate in Afrika das Niveau des Inlandsprodukts und dessen Wachstum? Begründen Sie Ihre Antwort mit Hilfe des Wachstumsmodells!

How does the high population growth rate in Africa influence the level of domestic product and its growth? Justify your answer with the help of the growth model!

Sample Solution: Eine hohe Bevölkerungswachstumsrate, wie sie viele Länder Afrikas aufweisen, impliziert, dass der gesamte Kapitalstock auf immer mehr Köpfe verteilt wird. Bei einem geringen Pro-Kopf-Kapitalstock stellt sich ein geringes Niveau des Pro-Kopf-Inlandsprodukts ein. Das Inlandsprodukt steigt aber stark aufgrund der hohen Bevölkerungswachstumsrate.

A high population growth rate, as is the case in many African countries, implies that the entire capital stock is distributed among more and more people. A low per capita capital stock results in a low level of per capita domestic product. However, the domestic product rises sharply due to the high population growth rate.

Question 4

Question: Für eine Zentralbank gelte der Goldstandard. Beschreiben Sie, was darunter zu verstehen ist und welche Konsequenzen sich für die Geldpolitik der Zentralbank daraus ergeben.

A central bank is on the gold standard. Describe what this means and the consequences for the central bank's monetary policy.

Sample Solution: Gilt für eine Zentralbank der Goldstandard, so muss die Zentralbank jederzeit ausgegebene Banknoten in Gold konvertieren können. Damit ist sie in der Emission von Banknoten exogen begrenzt, das Geldangebot ist also festgelegt. Änderungen in der Geldnachfrage führen folglich automatisch zu Änderungen des Zinses, auch wenn die Zentralbank den Zins stabil halten möchte.

If a central bank is on the gold standard, it must be able to convert the banknotes it issues into gold at any time. Thus, it is exogenously limited in the number of banknotes it can issue, i.e. the money supply is fixed. Changes in the demand for money therefore automatically lead to changes in the interest rate, even if the central bank wants to keep the interest rate stable.

Question 5

Question: Die mikrofundierte Makroökonomik erklärt das Konsumverhalten privater Haushalte mithilfe eines Optimierungskalküls bezogen auf das Lebenseinkommen. Wie würde sich, ausgehend von diesem Kalkül, eine einmalige Einkommenserhöhung (z.B. ein Lotteriegewinn) auf den Konsum eines Haushaltes auswirken?

Microfounded macroeconomics explains the consumption behavior of private households with the help of an optimization calculation based on lifetime income. Based on this calculation, how would a one-off increase in income (e.g. a lottery win) affect a household's consumption?

Sample Solution: Da das Einkommen eines Haushaltes in Abhängigkeit vom Lebensalter schwankt, wird in Zeiten hohen Einkommens gespart und in Zeiten von niedrigem (oder keinem) Einkommen entspart. Diese Konsumglättung geht laut Optimierungskalkül so weit, dass über alle Lebensphasen ein identisches Konsumniveau aufrechterhalten wird. Eine einmalige Einkommenserhöhung wird nach dieser Logik auf alle Lebensjahre verteilt und bewirkt daher nur eine sehr geringfügige Erhöhung des gegenwärtigen Konsums.

As a household's income fluctuates depending on age, people save in times of high income and dissave in times of low (or no) income. According to the optimization calculation, this smoothing of consumption goes so far that an identical level of consumption is maintained across all phases of life. According to this logic, a one-off increase in income is spread over all years of life and therefore only results in a very small increase in current consumption.

Question 6

Question: Was ist unter einem „automatischen Stabilisator" zu verstehen? Beantworten Sie die Frage für den Fall eines exogenen Anstiegs der Investitionen!

What is meant by an "automatic stabilizer"? Answer the question for the case of an exogenous increase in investment!

Sample Solution: Eine Erhöhung der Investitionen führt zu einer Zunahme des Inlandsprodukts (Boom). In welcher Höhe das Inlandsprodukt steigt, hängt vom Multiplikator ab. Erhebt der Staat Steuern, so ist der Multiplikator kleiner, als wenn der Staat keine Steuern erhebt. Durch die Steuererhebung wird also das Ausmaß des Booms automatisch reduziert und die gesamtwirtschaftliche Nachfrage stabilisiert, ohne dass der Staat spezielle Programme zur Dämpfung der Nachfrage auflegt.

An increase in investment leads to an increase in the domestic product (boom). The extent to which the domestic product increases depends on the multiplier. If the state levies taxes, the multiplier is lower than if the state does not levy taxes. Raising taxes therefore automatically reduces the extent of the boom and stabilizes overall economic demand without the state having to launch special programs to dampen demand.

S2 GPT-generated Answers

For each of the questions 2 –6, we generate five variants of GPT answers. These variants differ only in terms of the information that GPT receives to base its answer on:

- Variant 1: No information.
- Variant 2: Relevant textbook excerpt.
- Variant 3: Irrelevant textbook excerpt.
- Variant 4: Relevant and irrelevant textbook excerpt.
- Variant 5: 10 randomly selected student answers.

The replacement of student answers with GPT-generated answers is determined in a two-step procedure: In a first step, we determine whether there is a replacement of a student answer by a GPT-generated answer in each set of five student answers and if so, which of the five student answers is to be replaced. To do so, we draw a random integer between 0 and 6 for each of the ten sets per question. The drawn number indicates the position of the GPT-generated in the given set. For random draws of 0 or 6, no student answer in this set is replaced with a GPT-generated answer. A random draw of a 1 means that the first student answer in in the set is replaced by a GPT-generated answer, a random draw of a 2 means that the second student answer in the set is replaced by a GPT-generated answer, and so on. This procedure entails that at maximum one student answer per set can be replaced with a GPT-generated. The probability that a GPT-generated replaces a student answer in a set is therefore 5/7. In a second step, we then determine which of the five variants of GPT-generated answers is chosen as a replacement. Here, we draw a random integer between 1 and 5, where the numbers represent the variants 1 – 5 of GPT-generated answers. Hence, any variant can occur in more than one of the ten sets of the respective question. With this procedure, 39 student answers were replaced with GPT-generated answers across all questions.

Note: The sets for Question 1 contain no AI answers. The research question whether GPT might be biased towards GPT-generated answers was only developed after the human examiners had already performed the ranking and point assessment for Question 1.

Prompt (translated to English):

You are a student of macroeconomics and answer an open question by entering a text. Pay attention to precise wording and logical presentation.

Open question:
[OPEN QUESTION]

For variants 2, 3, and 4:

Please base your answer on the following textbook excerpt: [TEXTBOOK EXCERPT]

For variant 5:
Please base your answer on the following ten student answers: [STUDENT ANSWERS]

Answer:

Run Prompt

Shorten your answer to a maximum of [number based on average length of student answers] words:

Run Prompt

Add a typo to your answer:

Run Prompt

Prompt (Original Version in German):

Du bist eine Studentin der Makroökonomik und beantwortest eine offene Fragestellung durch Eingabe eines Textes. Achte auf präzise Formulierung und logische Darstellung.

Offene Fragestellung:

[OPEN QUESTION]

For variants 2, 3, and 4:

Bitte basiere deine Antwort auf folgendem Lehrbuchauszug:

[TEXTBOOK EXCERPT]

For variant 5:

Bitte basiere deine Antwort auf folgenden 10 Studierendenantworten:

[STUDENT ANSWERS]

Antwort:

Run Prompt

Kürze deine Antwort auf maximal [Zahl basierend auf mittlerer Länge der Studierendenantworten] Wörter:

Run Prompt

Füge deiner Antwort einen Tippfehler bei:

Run Prompt

S3 Instructions for Human Examiners

Instructions for Ranking Task (translated to English)

Please read the following instructions carefully before starting the task.

The goal of the task is to rank student responses to an open-ended question in macroeconomics based on a sample solution. It is important that you work on the task alone and independently of the others. You can of course talk during the task as usual, but please do not exchange ideas about the task and its contents. The others have received a similar task from us and are also asked to work on it alone and independently.

In the attachment of this e-mail you will find an Excel file. Once you open this file, you will see an Excel document with 10 spreadsheets: "Ranking1" to "Ranking10". At the top of each spreadsheet you will see an open question and the corresponding sample solution. These are the same on all spreadsheets. Below that, there are five randomly selected student answers. Your task is to assign a rank between 1 (best answer) and 5 (worst answer) for each student answer. Each rank can only be assigned once. Please consider the following criteria when assigning ranks: Correctness, completeness, and relevance to the question. You can base your ranking on the closeness of the student answer to the sample solution. Please enter the rank as a number next to the respective student answer in the column "Rank". If you consider two answers to be equivalent, you must still give preference to one. Use style and spelling to help you decide.

Once you have assigned all five ranks on a spreadsheet, move to the next spreadsheet. Once you have edited all ten spreadsheets, save the document as "Rangfolge1_erledigt" and email it to us. Please include an approximate time estimate in the email. This is for research purposes only.

Instructions for Ranking Task (Original in German)

Bitte lies die folgenden Instruktionen sorgfältig durch, bevor Du mit der Aufgabe beginnst.

Ziel der Aufgabe ist es, Rangfolgen von Studierendenantworten auf eine offene Frage im Fach Makroökonomie basierend auf einer Musterlösung zu bilden. Es ist wichtig, dass Du die Aufgabe alleine und unabhängig von den anderen bearbeitest. Ihr könnt Euch während der Aufgabe natürlich wie immer unterhalten, aber tauscht Euch bitte nicht über die Aufgabe und ihre Inhalte aus. Die anderen haben eine ähnliche Aufgabe von uns erhalten und sind auch gebeten, diese alleine und selbstständig zu bearbeiten.

Im Anhang dieser E-Mail befindet sich eine Excel-Datei. Sobald Du diese Datei öffnest, siehst Du ein Excel-Dokument mit 10 Tabellenblättern: „Ranking1“ bis „Ranking10“. Oben auf jedem Tabellenblatt siehst Du eine offene Frage und die dazugehörige Musterlösung. Diese sind auf allen Tabellenblättern gleich. Darunter befinden sich fünf zufällig ausgewählte Studierendenantworten. Deine Aufgabe ist es, für jede Studierendenantwort einen Rang zwischen 1 (beste Antwort) und 5 (schlechteste Antwort) zu vergeben. Jeder Rang kann nur ein Mal vergeben werden. Bitte beachte bei der Vergabe der Ränge folgende Kriterien: Richtigkeit, Vollständigkeit und Relevanz für die Fragestellung. Dabei kannst Du deine Rangvergabe auf der jeweiligen Nähe der Studierendenantwort zur Musterlösung basieren. Bitte trage den Rang neben der jeweiligen Studierendenantwort in der Spalte „Rang“ als Zahl ein. Falls Du zwei Antworten für gleichwertig hältst, musst Du dennoch eine bevorzugen. Ziehe Stil und Rechtschreibung als zusätzliche Entscheidungshilfen hinzu. Sobald Du alle fünf Ränge auf einem Tabellenblatt vergeben hast, wechsle auf das nächste Tabellenblatt.

Sobald Du alle zehn Tabellenblätter bearbeitet hast, speichere das Dokument unter „Rangfolge1_erledigt“ und sende es uns per E-Mail. Bitte mache in der Email eine ungefähre Angabe über den zeitlichen Aufwand. Dies dient lediglich zu Forschungszwecken.

Instructions for Point Assessment (translated to English)

Please read the following instructions carefully before starting the task.

The goal of this task is to award points for student answers to an open question in macroeconomics based on a sample solution. In the sample solution, the content for which a point is awarded is marked as follows: (1), (1 point) or (1P). The points (1), (1 point) or (1P) refer to the previous content.

In the attachment of this e-mail you will find an Excel file. Once you open this file, you will see an Excel document with 10 spreadsheets: "Ranking1" to "Ranking10". At the top of each spreadsheet you will see an open question and the corresponding sample solution. These are the same on all spreadsheets. Below that, there are five randomly selected student answers. Your task is to award a number of points for each student answer. 0 points are also possible. The sample solution contains all relevant and correct content for the question. It is complete. No more points can be awarded than in the sample solution. You can base your award of points on the proximity of the student's answer to the content of the sample solution. These are the relevant contents that are necessary for a correct and complete answer to the question. A score equal to the number of points awarded by the sample solution means that the student's answer contains all the relevant content of the sample solution. Only whole points can be awarded. Add up the points you have awarded for each student answer. Enter only the total number of points for each student answer.

Once you have assigned all five ranks on a spreadsheet, move to the next spreadsheet. Once you have edited all ten spreadsheets, save the document as "Bepunktung1_erledigt" and email it to us. Please include an approximate time estimate in the email. This is for research purposes only.

Instructions for Point Assessment Task (Original in German)

Bitte lies die folgenden Instruktionen sorgfältig durch, bevor Du mit der Aufgabe beginnst.

Ziel dieser Aufgabe ist es, für Studierendenantworten auf eine offene Frage im Fach Makroökonomik basierend auf einer Musterlösung Punkte zu vergeben. In der Musterlösung sind diejenigen Inhalte wie folgt gekennzeichnet, für die es einen Punkt gibt: (1), (1 Punkt) oder (1P). Die Bepunktungen (1), (1 Punkt) oder (1P) beziehen sich jeweils auf den vorausgegangenen Inhalt.

Im Anhang dieser E-Mail befindet sich eine Excel-Datei. Sobald Du diese Datei öffnest, siehst Du ein Excel-Dokument mit 10 Tabellenblättern: „Ranking1“ bis „Ranking10“. Oben auf jedem Tabellenblatt siehst Du eine offene Frage und die dazugehörige Musterlösung. Diese sind auf allen Tabellenblättern gleich. Darunter befinden sich fünf zufällig ausgewählte Studierendenantworten. Deine Aufgabe ist es, für jede Studierendenantwort eine Punktzahl zu vergeben. Auch 0 Punkte sind möglich. Die Musterlösung enthält alle für die Fragestellung relevante und richtige Inhalte. Sie ist vollständig. Es können nicht mehr Punkte vergeben werden als in der Musterlösung. Dabei kannst Du deine Vergabe der Punkte auf der jeweiligen Nähe der Studierendenantwort zu den bepunkteten Inhalten der Musterlösung basieren. Dies sind diejenigen relevanten Inhalte, die für die inhaltlich korrekte und vollständige Beantwortung der Frage notwendig sind. Eine Punktzahl in Höhe der von der Musterlösung vergebenen Punktzahl bedeutet, dass die Studierendenantwort alle relevanten Inhalte der Musterlösung enthält. Es können nur ganze Punkte vergeben werden. Addiere die jeweiligen Punkte, die du für eine Studierendenantwort vergeben hast. Gib für jede Studierendenantwort nur die jeweilige Gesamtpunktzahl an.

Sobald Du alle zehn Tabellenblätter bearbeitet hast, speichere das Dokument unter „Bepunktung1_erledigt“ und sende es uns per E-Mail. Bitte mache in der Email eine ungefähre Angabe über den zeitlichen Aufwand. Dies dient lediglich zu Forschungszwecken.

Note: The number in "Rangfolge1_erledigt" and "Bepunktung1_erledigt" indicates the question and is counted up to 6 with respect to the question at hand.

S4 Original Prompt

In this section, we present our original prompt, which is originally in German, alongside its English translation.

English Translation

Supplementary Table 1. Zero shot prompt template utilized in our experiments for investigating H1 w.r.t ranking.

You are a student assistant for macroeconomics and are given a task. The aim of this task is to rank student answers to an open question in macroeconomics based on a sample solution.<br><br>This is the open question:<br><br>[INSERT_QUESTION]<br><br>This is the sample solution:<br><br>[INSERT_SAMPLE_SOLUTION]<br><br>These are five randomly selected student answers in random order:<br><br>Student answer_1: [STUDENT_ANSWER_1]<br><br>Student answer_2: [STUDENT_ANSWER_2]<br><br>Student answer_3: [STUDENT_ANSWER_3]<br><br>Student answer_4: [STUDENT_ANSWER_4]<br><br>Student answer_5: [STUDENT_ANSWER_5]<br><br>Your task is to assign a rank between 1 (best answer) and 5 (worst answer) for each student answer. Each rank can only be awarded once. Please consider the following criteria when assigning ranks: Correctness, completeness and relevance to the question. You can base your ranking on the proximity of the student's answer to the sample solution. If you consider two answers to be of equal value, you must still give preference to one. Use style and spelling as additional decision-making criteria.<br><br>Present your ranking as follows (state only the answer and the corresponding rank):<br><br>Student answer_X: Rank Y<br><br>Output:\n

Supplementary Table 2. Zero shot prompt template utilized in our experiments for investigating H1 w.r.t point assessment.

You are a student assistant for macroeconomics and are given a task. The aim of this task is to award points for student answers to an open question in macroeconomics based on a sample solution. In the sample solution, the content for which a point is awarded is marked as follows: (1), (1 point) or (1P). The points (1), (1 point) or (1P) refer to the previous content.

This is the open question:

[INSERT_QUESTION]

This is the sample solution:

[INSERT_SAMPLE_SOLUTION]

These are five randomly selected student answers in random order:

Student answer_1: [STUDENT_ANSWER_1]

Student answer_2: [STUDENT_ANSWER_2]

Student answer_3: [STUDENT_ANSWER_3]

Student answer_4: [STUDENT_ANSWER_4]

Student answer_5: [STUDENT_ANSWER_5]

Your task is to award a number of points for each student answer. 0 points are also possible. The sample solution contains all relevant and correct content for the question. It is complete. No more points can be awarded than in the sample solution.

You can base your award of points on the respective proximity of the student's answer to the points awarded for the content of the sample solution. This is the relevant content that is necessary to answer the question correctly and completely. A score equal to the number of points awarded by the sample solution means that the student's answer contains all the relevant content of the sample solution. Only whole points can be awarded. Add up the points you have awarded for each student answer. State only the total number of points for each student answer.

Present your point assessment as follows (state only the answer and the number of points awarded):

Student answer_X: Points

Output:\n

German (original)

Supplementary Table 3. Zero shot prompt template utilized in our experiments for investigating H1 w.r.t ranking.

Du bist eine studentische Hilfskraft für das Fach Makroökonomik und bekommst eine Aufgabe gestellt. Ziel dieser Aufgabe ist es, eine Rangfolge von Studierendenantworten auf eine offene Frage im Fach Makroökonomik basierend auf einer Musterlösung zu bilden.<br><br>Dies ist die offene Frage:<br><br>[INSERT_FRAGE]<br><br>Dies ist die Musterlösung:<br><br>[INSERT_SAMPLE_SOLUTION]<br><br>Dies sind fünf zufällig ausgewählte Studierendenantworten in zufälliger Reihenfolge:<br><br>Studierendenantwort_1: [STUDENT_ANSWER_1]<br><br>Studierendenantwort_2: [STUDENT_ANSWER_2]<br><br>Studierendenantwort_3: [STUDENT_ANSWER_3]<br><br>Studierendenantwort_4: [STUDENT_ANSWER_4]<br><br>Studierendenantwort_5: [STUDENT_ANSWER_5]<br><br>Deine Aufgabe ist es, für jede Studierendenantwort einen Rang zwischen 1 (beste Antwort) und 5 (schlecht-este Antwort) zu vergeben. Jeder Rang kann nur ein Mal vergeben werden. Bitte beachte bei der Vergabe der Rängefolgende Kriterien: Richtigkeit, Vollständigkeit und Relevanz für die Fragestellung. Dabei kannst Du deine Rangvergabeauf der jeweiligen Nähe der Studierendenantwort zur Musterlösung basieren. Falls Du zwei Antworten für gleichwer-tig hältst, musst Du dennoch eine bevorzugen. Ziehe Stil und Rechtschreibung als zusätzliche Entscheidungshilfen hinzu.<br><br>Stelle deine Rangvergabe wie folgt dar (nenne nur die Antwort und den dazugehörigen Rang):<br><br>Studierendenantwort_X: Rang Y<br><br>Output:\n

Supplementary Table 4. Zero shot prompt template utilized in our experiments for investigating H1 w.r.t point assessment.

Du bist eine studentische Hilfskraft für das Fach Makroökonomik und bekommst eine Aufgabe gestellt. Ziel dieser Aufgabe ist es, für Studierendenantworten auf eine offene Frage im Fach Makroökonomik basierend auf einer Musterlösung Punkte zu vergeben. In der Musterlösung sind diejenigen Inhalte wie folgt gekennzeichnet, für die es einen Punkt gibt: (1), (1 Punkt) oder (1P). Die Bepunktungen (1), (1 Punkt) oder (1P) beziehen sich jeweils auf den vorausgegangenen Inhalt.<br><br>Dies ist die offene Frage:<br><br>[INSERT_FRAGE]<br><br>Dies ist die Musterlösung:<br><br>[INSERT_SAMPLE_SOLUTION]<br><br>Dies sind fünf zufällig ausgewählte Studierendenantworten in zufälliger Reihenfolge:<br><br>Studierendenantwort_1: [STUDENT_ANSWER_1]<br><br>Studierendenantwort_2: [STUDENT_ANSWER_2]<br><br>Studierendenantwort_3: [STUDENT_ANSWER_3]<br><br>Studierendenantwort_4: [STUDENT_ANSWER_4]<br><br>Studierendenantwort_5: [STUDENT_ANSWER_5]<br><br>Deine Aufgabe ist es, für jede Studierendenantwort eine Punktzahl zu vergeben. Auch 0 Punkte sind möglich. Die Musterlösung enthält alle für die Fragestellung relevante und richtige Inhalte. Sie ist vollständig. Es können nicht mehr Punkte vergeben werden als in der Musterlösung.<br><br>Dabei kannst Du deine Vergabe der Punkte auf der jeweiligen Nähe der Studierendenantwort zu den bepunk-teten Inhalten der Musterlösung basieren. Dies sind diejenigen relevanten Inhalte, die für die inhaltlich korrekte undvollständige Beantwortung der Frage notwendig sind. Eine Punktzahl in Höhe der von der Musterlösung vergebenenPunktzahl bedeutet, dass die Studierendenantwort alle relevanten Inhalte der Musterlösung enthält. Es können nurganze Punkte vergeben werden. Addiere die jeweiligen Punkte, die du für eine Studierendenantwort vergeben hast. Gibfür jede Studierendenantwort nur die jeweilige Gesamtpunktzahl an.<br><br>Stelle deine Rangvergabe wie folgt dar (nenne nur die Antwort und die vergebene Punktzahl):<br><br>Studierendenantwort_X: Punktzahl<br><br>Output:\n

S5 Extended Results for Rank and Point Assessments addressing Hypothesis 1

This section presents the extended result for rank assessment in Supplementary Table 5 and point assessment in Table 6.

Supplementary Table 5. Summary statistics for Inter-rater reliability using Kendall's W for all questions in rank assessment

Questions	Human team	AI-human team 1	AI-human team 2	AI-human team 3
Question 1	$0.791^{\pm 0.107}$	$0.838^{\pm 0.070}$	$0.858^{\pm 0.073}$	$0.824^{\pm 0.078}$
Question 2	$0.899^{\pm 0.051}$	$0.859^{\pm 0.085}$	$0.858^{\pm 0.093}$	$0.879^{\pm 0.096}$
Question 3	$0.793^{\pm 0.176}$	$0.716^{\pm 0.168}$	$0.771^{\pm 0.135}$	$0.707^{\pm 0.153}$
Question 4	$0.824^{\pm 0.104}$	$0.856^{\pm 0.067}$	$0.869^{\pm 0.053}$	$0.811^{\pm 0.104}$
Question 5	$0.776^{\pm 0.149}$	$0.780^{\pm 0.123}$	$0.804^{\pm 0.158}$	$0.787^{\pm 0.108}$
Question 6	$0.671^{\pm 0.191}$	$0.684^{\pm 0.199}$	$0.676^{\pm 0.150}$	$0.716^{\pm 0.181}$
Average	$0.792^{\pm 0.149}$	$0.789^{\pm 0.141}$	$0.806^{\pm 0.131}$	$0.787^{\pm 0.134}$

Standard deviation provided as superscript

Supplementary Table 6. Summary statistics for Inter-rater reliability using Cronbach's Alpha for all questions in point assessment

Questions	Human team	AI-human team 1	AI-human team 2	AI-human team 3
Question 1	0.935 [0.896, 0.961]	0.914 [0.862, 0.948]	0.896 [0.833, 0.937]	0.908 [0.852, 0.945]
Question 2	0.938 [0.901, 0.963]	0.943 [0.909, 0.966]	0.943 [0.908, 0.966]	0.936 [0.898, 0.962]
Question 3	0.925 [0.880, 0.955]	0.690 [0.504, 0.814]	0.724 [0.558, 0.834]	0.735 [0.576, 0.841]
Question 4	0.918 [0.869, 0.951]	0.924 [0.878, 0.954]	0.936 [0.897, 0.961]	0.920 [0.873, 0.952]
Question 5	0.904 [0.847, 0.943]	0.874 [0.798, 0.924]	0.927 [0.883, 0.956]	0.873 [0.797, 0.924]
Question 6	0.903 [0.844, 0.941]	0.895 [0.832, 0.937]	0.844 [0.751, 0.906]	0.869 [0.791, 0.921]
Pooled	0.944 [0.932, 0.954]	0.933 [0.918, 0.945]	0.932 [0.917, 0.944]	0.933 [0.919, 0.945]

Confidence Interval at 95% provided in square brackets

S6 Permutation Tests

Differences between the mean values of GPT-4 and the three human examiners when awarding points were tested for significance using a permutation test. Parametric tests were deemed unsuitable due to differences in the underlying distributions of the data. For each pairwise test, we used an array of 300 observations from GPT-4 and 300 observations from one selected human examiner. A joint array of all 600 observations was constructed. We then randomly divided these 600 observations into two sets of 300 values. For each random division, we calculated the difference in mean values between the two sets. This process was repeated 10,000 times, generating a distribution of differences. We then compared the observed difference in means between GPT-4 and the human examiner to this distribution, determining the proportion of simulated differences that were equal to or greater than the observed mean. This proportion reflects the probability of observing such a difference by chance. In all cases, this probability was below 0.1%, indicating a statistically significant difference between GPT-4 and each of the three human examiners. For further details on permutation tests, see Efron and Tibshirani (1994) [1].

We applied a similar procedure to test the significance of differences between pairwise QWKs (ranking) as well as Pearson correlations (point assessment). For example, the Pearson correlation between humans 1 and 2 is 0.861, while the Pearson correlation between human 1 and GPT-4 is 0.795. To determine whether the observed difference of 0.861 - 0.795 = 0.066 is statistically significant, we constructed a matrix with two rows and 600 columns. The first row contained the data for human 1 repeated twice, while the second row combined the data for human 2 (first 300 columns) and GPT-4 (last 300 columns). A split after column 300 reconstructs the original pairings, allowing us to compute the correlation between rows and reproduce the observed data. To test significance, we randomly reassigned the 600 columns into two sets of 300 columns. During the randomization, each column was treated as a unit, preserving the pair of values in row 1 and row 2 for that column. For each random division, we computed the correlation between rows for both sets and calculated the difference between the two correlations. This process was repeated 10,000 times, generating a distribution of differences under the null hypothesis. Finally, we compared the observed difference in correlations of 0.066 to this distribution to calculate our test statistic. In this case, with $p=0.028$, we infer that the observed difference is unlikely to occur by chance.

For the joint test on whether point assessments by humans correlate better with each other than with GPT-4, we first created an expanded set of 900 observations combining data from humans 1, 2, and 3. A second set was constructed by rearranging the sequence of these observations to human 2, 3, and 1. These were organized into a matrix with two rows and 900 columns. The correlation between the two rows was 0.847. Next, we created a comparable dataset for GPT-4 by repeating its 300 observations three times to achieve a length of 900. A second matrix was formed with the first row containing the combined human data (humans 1, 2, and 3) and the second row containing the repeated GPT-4 data. The correlation between these rows was lower, at 0.812. To test whether the observed difference of 0.847 - 0.812 = 0.035 is statistically significant, we used the procedure described for single correlation tests. Specifically, we created a joint matrix with two rows and 1800 columns, combining both matrices. Across 10,000 iterations, we randomly split this joint matrix into two sets, each with two rows and 900 columns, treating each column as a unit. For each split, we computed the correlation between rows for both sets and calculated the difference in correlations. This process generated a distribution of differences under the null hypothesis. Finally, we compared the observed difference of 0.035 to this distribution. With a $p=0.024$, we conclude that the observed difference is unlikely to occur by chance, indicating a statistically significant result.

[1] Efron, B., & Tibshirani, R. J. (1994). An introduction to the bootstrap. Chapman and Hall/CRC.

S7 Single Answer Point Assessment Prompt

Supplementary Table 7. Single Answer Point Assessment Prompt (original version in German)

Du bist eine studentische Hilfskraft für das Fach Makroökonomik und bekommst eine Aufgabe gestellt. Ziel dieser Aufgabe ist es, für eine Studierendenantwort auf eine offene Frage im Fach Makroökonomik basierend auf einer Musterlösung Punkte zu vergeben. In der Musterlösung sind diejenigen Inhalte wie folgt gekennzeichnet, für die es einen Punkt gibt: (1), (1 Punkt) oder (1P). Die Bepunktungen (1), (1 Punkt) oder (1P) beziehen sich jeweils auf den vorausgegangenen Inhalt.

Dies ist die offene Frage:

[INSERT_FRAGE]

Dies ist die Musterlösung:

[INSERT_MASTERSOLUTION]

Dies ist die Studierendenantwort:

[STUDENT_ANSWER]

Deine Aufgabe ist es, für die Studierendenantwort eine Punktzahl zu vergeben. Auch 0 Punkte sind möglich. Die Musterlösung enthält alle für die Fragestellung relevante und richtige Inhalte. Sie ist vollständig. Es können nicht mehr Punkte vergeben werden als in der Musterlösung.

Dabei kannst du deine Vergabe der Punkte auf der jeweiligen Nähe der Studierendenantwort zu den bepunkteten Inhalten der Musterlösung basieren. Dies sind diejenigen relevanten Inhalte, die für die inhaltlich korrekte und vollständige Beantwortung der Frage notwendig sind. Eine Punktzahl in Höhe der von der Musterlösung vergebenen Punktzahl bedeutet, dass die Studierendenantwort alle relevanten Inhalte der Musterlösung enthält. Es können nur ganze Punkte vergeben werden. Addiere die jeweiligen Punkte, die du für eine Studierendenantwort vergeben hast. Gib für die Studierendenantwort nur die jeweilige Gesamtpunktzahl an.

Stelle deine Rangvergabe wie folgt dar (nenne nur die Antwort und die vergebene Punktzahl):

Studierendenantwort: X Punkte

Output:\n

Supplementary Table 8. Single Answer Point Assessment Prompt (English translation)

You are a student assistant for the course Macroeconomics and are given a task. The goal of this task is to assign points to a student's answer to an open question in Macroeconomics based on a sample solution. In the sample solution, the contents for which points are awarded are marked as follows: (1), (1 point), or (1P). The markings (1), (1 point), or (1P) each refer to the preceding content.<br><br>This is the open question:<br><br>[INSERT_QUESTION]<br><br>This is the sample solution:<br><br>[INSERT_MASTERSOLUTION]<br><br>This is the student answer:<br><br>[STUDENT_ANSWER]<br><br>Your task is to assign a score to the student's answer. Zero points are also possible. The sample solution contains all relevant and correct contents for the question. It is complete. It is not possible to assign more points than in the sample solution.<br><br>You can base your award of points on the respective proximity of the student's answer to the points awarded for the content of the sample solution. This is the relevant content that is necessary to answer the question correctly and completely. A score equal to the number of points awarded by the sample solution means that the student's answer contains all the relevant content of the sample solution. Only whole points can be awarded. Add up the points you have awarded for each student answer. State only the total number of points for each student answer. Only whole points can be awarded. Add the respective points you have assigned to a student answer. Indicate only the total score for the student answer.<br><br>Present your scoring as follows (name only the answer and the awarded score):<br><br>Student answer: Score<br><br>Output:\n

S8 Additional Results Length Bias

	(1) Single Answer	(2) Below Median	(3) Above/Equal Median	(4) Two-Step Reasoning	(5) Additional Instructions
Human Examiner 1	0.37***	0.70***	0.27*	0.22*	0.32***
	(0.1)	(0.2)	(0.2)	(0.1)	(0.1)
Human Examiner 2	0.74***	0.94***	0.61***	0.75***	0.67***
	(0.1)	(0.2)	(0.2)	(0.1)	(0.1)
Human Examiner 3	0.77***	0.80***	0.85***	0.52***	0.81***
	(0.1)	(0.2)	(0.2)	(0.1)	(0.1)
Length	0.037***	0.025	0.046***	0.019**	0.033***
	(0.01)	(0.03)	(0.02)	(0.01)	(0.01)
GPT_Answer	0.27	0.45	0.40	-0.33	0.037
	(0.2)	(0.3)	(0.5)	(0.2)	(0.2)
Task controls	Yes	Yes	Yes	Yes	Yes
Observations	300	156	144	300	300
Pseudo R^2	0.49	0.53	0.46	0.42	0.48

Standard errors in parentheses
* $p < 0.10$, ** $p < 0.05$, *** $p < 0.01$

Column (1) reports the single-answer results, which serve as the reference case. Columns (2) and (3) show the results under a median split: column (2) for answers shorter or equal to the median length of 277 characters, and column (3) for answers longer than 277 characters. Comparing (2) and (3) with (1), we can see that the length bias is smaller and statistically insignificant for shorter answers, but higher and significant (p < 0.01) for longer ones. This suggests that length bias is primarily driven by longer answers, implying that restricting the maximum number of characters could help mitigate it.

Columns (4) and (5) present results from two alternative prompting strategies aimed at reducing the impact of answer length on GPT's assessment. In column (4), we use a two-step reasoning prompt. First, the LLM scans the student answer for all content-relevant aspects of the sample solution, determining for each whether it is correctly, completely, and clearly present ("yes" or "no"). Second, it counts the total number of "yes" and "no" decisions, outputting both the list of aspects with their labels and the total number of "yes" points. This method reduces the point coefficient from 0.037 to 0.019, though length remains a significant predictor of GPT's scores (p<0.05).

The prompt in column (5) adds explicit instructions to ignore answer length to our original prompt, stating that length should neither positively nor negatively influence the assessment. This approach only marginally reduces the length bias compared to the original prompt in column (1); it again remains significant. The corresponding German and English translated prompts for (4) and (5) are provided in Supplementary Table 9, 10 and Table 11, 12, respectively.

Supplementary Table 9. Two-step reasoning prompt (original version in German)

Du bist eine studentische Hilfskraft für das Fach Makroökonomik und bekommst eine Aufgabe gestellt. Ziel dieser Aufgabe ist es, für eine Studierendenantwort auf eine offene Frage im Fach Makroökonomik basierend auf einer Musterlösung Punkte zu vergeben. In der Musterlösung sind diejenigen Inhalte wie folgt gekennzeichnet, für die es einen Punkt gibt: (1), (1 Punkt) oder (1P). Die Bepunktungen (1), (1 Punkt) oder (1P) beziehen sich jeweils auf den vorausgegangenen Inhalt.<br><br>Dies ist die offene Frage:<br><br>[INSERT_FRAGE]<br><br>Dies ist die Musterlösung:<br><br>[INSERT_MASTERSOLUTION]<br><br>Dies ist die Studierendenantwort:<br><br>[STUDENT_ANSWER]<br><br>Deine Aufgabe ist es, für die Studierendenantwort eine Punktzahl zu vergeben. Auch 0 Punkte sind möglich. Die Musterlösung enthält alle für die Fragestellung relevante und richtige Inhalte. Sie ist vollständig. Es können nicht mehr Punkte vergeben werden als in der Musterlösung.<br><br>Dabei kannst du deine Vergabe der Punkte auf der jeweiligen Nähe der Studierendenantwort zu den bepunkteten Inhalten der Musterlösung basieren. Dies sind diejenigen relevanten Inhalte, die für die inhaltlich korrekte und vollständige Beantwortung der Frage notwendig sind. Eine Punktzahl in Höhe der von der Musterlösung vergebenen Punktzahl bedeutet, dass die Studierendenantwort alle relevanten Inhalte der Musterlösung enthält.<br><br>Gehe bei der Bewertung immer in zwei Schritten vor:<br><br>Schritt 1: Liste alle bepunkteten Inhalte aus der Musterlösung auf und gib für jeden Punkt an, ob dieser Inhalt eindeutig, vollständig und korrekt in der Studierendenantwort enthalten ist („Ja“ oder „Nein“). Ein Punkt darf nur vergeben werden, wenn die entsprechende Aussage in der Studierendenantwort inhaltlich gleichwertig zur Musterlösung ist. Dabei muss die Aussage inhaltlich korrekt und vollständig erfasst sein, auch wenn sie in anderen Worten formuliert ist. Wird ein Punkt inkorrekt, unvollständig, nur vage oder unklar angesprochen, darf er nicht berücksichtigt werden. Verwende bei der Auflistung der bepunkteten Inhalte genau die Formulierungen aus der Musterlösung, ohne sie zu kürzen oder umzuformulieren.<br><br>Schritt 2: Zähle die Punkte, deren Inhalte in Schritt 1 als „Ja“ markiert wurden.<br><br>Gib die Bewertung ausschließlich wie folgt aus:<br><br>Liste der bepunkteten Inhalte mit Ja/Nein aus Schritt 1: [Inhalt 1]: Ja/Nein [Inhalt 2]: Ja/Nein<br><br>Gesamte Punktzahl aus Schritt 2: Studierendenantwort: X Punkte<br><br>Output:\n

Supplementary Table 10. Two-step reasoning prompt (English translation)

You are a student assistant for the Macroeconomics course and have been given a task. The goal of this task is to assign points to a student's answer to an open question in Macroeconomics based on a sample solution. In the sample solution, the contents for which points are awarded are marked as follows: (1), (1 point), or (1P). These point markings (1), (1 point), or (1P) each refer to the preceding content.<br><br>This is the open question:<br><br>[INSERT_QUESTION]<br><br>This is the sample solution:<br><br>[INSERT_MASTERSOLUTION]<br><br>This is the student answer:<br><br>[STUDENT_ANSWER]<br><br>Your task is to assign a score to the student's answer. Zero points are also possible. The sample solution contains all relevant and correct content for the question. It is complete. It is not possible to assign more points than in the sample solution.<br><br>You can base your point allocation on how closely the student's answer corresponds to the point-awarded contents of the sample solution. These are the relevant contents necessary for a correct and complete answer to the question. A score equal to the points awarded by the sample solution means the student answer contains all relevant contents of the sample solution.<br><br>Always proceed in two steps when grading:<br><br>Step 1: List all point-awarded contents from the sample solution and indicate for each point whether this content is clearly, completely, and correctly contained in the student answer ("Yes" or "No"). A point may only be awarded if the corresponding statement in the student answer is substantively equivalent to the sample solution. The statement must be factually correct and complete, even if expressed in different words. If a point is addressed incorrectly, incompletely, vaguely, or unclearly, it must not be counted. When listing the point-awarded contents, use exactly the formulations from the sample solution without shortening or rephrasing them.<br><br>Step 2: Count the points whose contents were marked as "Yes" in Step 1.<br><br>Give the evaluation output exclusively as follows:<br><br>List of point-awarded contents with Yes/No from Step 1: [Content 1]: Yes/No [Content 2]: Yes/No<br><br>Total points from Step 2: Student answer: X points<br><br>Output:\n

Supplementary Table 11. Original prompt with additional instructions to disregard length (original version in German)

Du bist eine studentische Hilfskraft für das Fach Makroökonomik und bekommst eine Aufgabe gestellt. Ziel dieser Aufgabe ist es, für eine Studierendenantwort auf eine offene Frage im Fach Makroökonomik basierend auf einer Musterlösung Punkte zu vergeben. In der Musterlösung sind diejenigen Inhalte wie folgt gekennzeichnet, für die es einen Punkt gibt: (1), (1 Punkt) oder (1P). Die Bepunktungen (1), (1 Punkt) oder (1P) beziehen sich jeweils auf den vorausgegangenen Inhalt.<br><br>Dies ist die offene Frage:<br><br>[INSERT_QUESTION]<br><br>Dies ist die Musterlösung:<br><br>[INSERT_MASTERSOLUTION]<br><br>Dies ist die Studierendenantwort:<br><br>[STUDENT_ANSWER]<br><br>Deine Aufgabe ist es, für die Studierendenantwort eine Punktzahl zu vergeben. Auch 0 Punkte sind möglich. Die Musterlösung enthält alle für die Fragestellung relevante und richtige Inhalte. Sie ist vollständig. Es können nicht mehr Punkte vergeben werden als in der Musterlösung.<br><br>Dabei kannst du deine Vergabe der Punkte auf der jeweiligen Nähe der Studierendenantwort zu den bepunkteten Inhalten der Musterlösung basieren. Dies sind diejenigen relevanten Inhalte, die für die inhaltlich korrekte und vollständige Beantwortung der Frage notwendig sind. Eine Punktzahl in Höhe der von der Musterlösung vergebenen Punktzahl bedeutet, dass die Studierendenantwort alle relevanten Inhalte der Musterlösung enthält. Beachte bei deiner Bewertung immer folgenden Grundsatz: Die Musterlösung enthält alle relevanten Inhalte, dient jedoch nicht als Maßstab für die Länge. Die Länge der Antwort darf nicht die Bewertung beeinflussen. Die Studierendenantwort kann unabhängig von ihrer Länge die volle Punktzahl erhalten, sofern sie alle relevanten Inhalte vollständig und korrekt enthält. Irrelevante, wiederholte oder ausschweifende Inhalte dürfen die Punktzahl nicht erhöhen. Die Länge der Antwort darf niemals – weder positiv noch negativ – die Punktevergabe beeinflussen.<br><br>Es können nur ganze Punkte vergeben werden. Addiere die jeweiligen Punkte, die du für eine Studierendenantwort vergeben hast. Gib für die Studierendenantwort nur die jeweilige Gesamtpunktzahl an.<br><br>Stelle deine Rangvergabe wie folgt dar (nenne nur die Antwort und die vergebene Punktzahl):<br><br>Studierendenantwort: Punktzahl<br><br>Output:\n

Supplementary Table 12. Original prompt with additional instructions to disregard length (English translation)

You are a student assistant for the course Macroeconomics and are given a task. The goal of this task is to assign points to a student's answer to an open question in Macroeconomics based on a sample solution. In the sample solution, the contents for which points are awarded are marked as follows: (1), (1 point), or (1P). The markings (1), (1 point), or (1P) each refer to the preceding content.<br><br>This is the open question:<br><br>[INSERT_QUESTION]<br><br>This is the sample solution:<br><br>[INSERT_MASTERSOLUTION]<br><br>This is the student answer:<br><br>[STUDENT_ANSWER]<br><br>Your task is to assign a score to the student's answer. Zero points are also possible. The sample solution contains all relevant and correct contents for the question. It is complete. It is not possible to assign more points than in the sample solution.<br><br>You can base your scoring on how closely the student's answer matches the point-awarded contents of the sample solution. These are the relevant contents necessary for a correct and complete answer to the question. A score equal to the points awarded by the sample solution means that the student's answer contains all relevant contents of the sample solution. Always keep the following principle in mind when grading: The sample solution contains all relevant contents, but it does not serve as a standard for length. The length of the answer must not influence the evaluation. The student's answer can receive full points regardless of its length, provided it contains all relevant contents fully and correctly. Irrelevant, repeated, or rambling content must not increase the score. The length of the answer must never—neither positively nor negatively—affect the awarding of points.<br><br>Only whole points can be awarded. Add the respective points you have assigned to a student answer. Indicate only the total score for the student answer.<br><br>Present your scoring as follows (name only the answer and the awarded score):<br><br>Student answer: Score<br><br>Output:\n

S9 Robustness Checks and Extensions

This section offers the English translation of the prompt in the Robustness Checks and extensions section regarding rank and point assessment.

Regarding, Prompt $P_{v6.1}$ and Prompt $P_{v6.2}$, for both rank and point assessment, since they deal with *spelling errors* in the original German prompt, we cannot provide a translation since the spelling errors cannot be reproduced through translation. We provide the German prompt as it is for rank assessment in Supplementary Table 17 and 18 and for point assessment in Supplementary Table 29 and 30.

S9.1 Prompts for Rank Assessment (English Translation)

Supplementary Table 13. Prompt P_{v2} (order of instruction) - Rank assessment

You are a student assistant for macroeconomics and are given a task. The aim of this task is to rank student answers to an open question in macroeconomics based on a sample solution.<br><br>Your task is to assign a rank between 1 (best answer) and 5 (worst answer) for each student answer. Each rank can only be awarded once. Please consider the following criteria when assigning ranks: Correctness, completeness and relevance to the question. You can base your ranking on the proximity of the student's answer to the sample solution. If you consider two answers to be of equal value, you must still give preference to one. Use style and spelling as additional decision-making criteria.<br><br>This is the open question:<br><br>[INSERT_QUESTION]<br><br>This is the sample solution:<br><br>[INSERT_SAMPLE_SOLUTION]<br><br>These are five randomly selected student answers in random order:<br><br>Student answer_1: [STUDENT_ANSWER_1]<br><br>Student answer_2: [STUDENT_ANSWER_2]<br><br>Student answer_3: [STUDENT_ANSWER_3]<br><br>Student answer_4: [STUDENT_ANSWER_4]<br><br>Student answer_5: [STUDENT_ANSWER_5]<br><br>Present your ranking as follows (state only the answer and the corresponding rank):<br><br>Student answer_X: Rank Y<br><br>Output:\n

Supplementary Table 14. Prompt P_{v3} (order of instruction) - Rank assessment

This is the open question:

[INSERT_QUESTION]

This is the sample solution:

[INSERT_SAMPLE_SOLUTION]

These are five randomly selected student answers in random order:

Student answer_1: [STUDENT_ANSWER_1]

Student answer_2: [STUDENT_ANSWER_2]

Student answer_3: [STUDENT_ANSWER_3]

Student answer_4: [STUDENT_ANSWER_4]

Student answer_5: [STUDENT_ANSWER_5]

You are a student assistant for macroeconomics and are given a task. The aim of this task is to rank student answers to an open question in macroeconomics based on a sample solution.

Your task is to assign a rank between 1 (best answer) and 5 (worst answer) for each student answer. Each rank can only be awarded once. Please consider the following criteria when assigning ranks: Correctness, completeness and relevance to the question. You can base your ranking on the proximity of the student's answer to the sample solution. If you consider two answers to be of equal value, you must still give preference to one. Use style and spelling as additional decision-making criteria.

Present your ranking as follows (state only the answer and the corresponding rank):

Student answer_X: Rank Y

Output:\n

Supplementary Table 15. Prompt P_{v4} (order of instruction) - Rank assessment

You are a student assistant for macroeconomics and are given a task. The aim of this task is to rank student answers to an open question in macroeconomics based on a sample solution.<br><br>This is the sample solution:<br><br>[INSERT_SAMPLE_SOLUTION]<br><br>This is the open question:<br><br>[INSERT_QUESTION]<br><br>These are five randomly selected student answers in random order:<br><br>Student answer_1: [STUDENT_ANSWER_1]<br><br>Student answer_2: [STUDENT_ANSWER_2]<br><br>Student answer_3: [STUDENT_ANSWER_3]<br><br>Student answer_4: [STUDENT_ANSWER_4]<br><br>Student answer_5: [STUDENT_ANSWER_5]<br><br>Your task is to assign a rank between 1 (best answer) and 5 (worst answer) for each student answer. Each rank can only be awarded once. Please consider the following criteria when assigning ranks: Correctness, completeness and relevance to the question. You can base your ranking on the proximity of the student's answer to the sample solution. If you consider two answers to be of equal value, you must still give preference to one. Use style and spelling as additional decision-making criteria.<br><br>Present your ranking as follows (state only the answer and the corresponding rank):<br><br>Student answer_X: Rank Y<br><br>Output:\n

Supplementary Table 16. Prompt P_{v5} (order of instruction) - Rank assessment

You are a student assistant for macroeconomics and are given a task. The aim of this task is to rank student answers to an open question in macroeconomics based on a sample solution.<br><br>This is the open question:<br><br>[INSERT_QUESTION]<br><br>These are five randomly selected student answers in random order:<br><br>Student answer_1: [STUDENT_ANSWER_1]<br><br>Student answer_2: [STUDENT_ANSWER_2]<br><br>Student answer_3: [STUDENT_ANSWER_3]<br><br>Student answer_4: [STUDENT_ANSWER_4]<br><br>Student answer_5: [STUDENT_ANSWER_5]<br><br>This is the sample solution:<br><br>[INSERT_SAMPLE_SOLUTION]<br><br>Your task is to assign a rank between 1 (best answer) and 5 (worst answer) for each student answer. Each rank can only be awarded once. Please consider the following criteria when assigning ranks: Correctness, completeness and relevance to the question. You can base your ranking on the proximity of the student's answer to the sample solution. If you consider two answers to be of equal value, you must still give preference to one. Use style and spelling as additional decision-making criteria.<br><br>Present your ranking as follows (state only the answer and the corresponding rank):<br><br>Student answer_X: Rank Y<br><br>Output:\n

Supplementary Table 17. Prompt $P_{v6.1}$ (major spelling error) - Rank assessment

Du bhist eiene studäntische Hilfskraft führ dass Fach Makroökonomik unt bekommst einne Aufgabbe gestellet. Zihl dieser Aufgabee ist es, einne Rangvolge von Studirendenantworten auf einne offene Ffrage im Fach Makroökonomek basirend auf einer Musterlözung zu bilden.<br><br>Diss ist dei offenne Ffrage:<br><br>[INSERT_FRAGE]<br><br>Diss ist die Musterlössung:<br><br>[INSERT_SAMPLE_SOLUTION]<br><br>Diss sind fünv zufälig ausgevvälte Studierendenantworten in zufäliger Reihenfolgee:<br><br>Studierendenantwort_1: [STUDENT_ANSWER_1]<br><br>Studierendenantwort_2: [STUDENT_ANSWER_2]<br><br>Studierendenantwort_3: [STUDENT_ANSWER_3]<br><br>Studierendenantwort_4: [STUDENT_ANSWER_4]<br><br>Studierendenantwort_5: [STUDENT_ANSWER_5]<br><br>Deinne Aufgabee ist es, für jede Studierendennantwort einen Rrang zwisschen 1 (bäste Antwort) und 5 (schlechteste Antwort) zu vergeben. Jeder Rang kann nur ein Mall vergeben werdden. Bittte beachte bei der Vergabe der Ränge folgende Kriterrien: Richtigkheit, Vollständigkheit und Relevanz für die Fragestelung. Dabei kkanst Du deine Rangvergabe auf der jeweiligen Nähe der Studierendennantwort zur Musterlösung basieren. Falls Du zwei Antwotten für gleichwertig hältst, must Du dennoch eine bevorzugen. Ziee Stil und Rechtschreibung als zusätzliche Entscheidungshilfen hinzuh.<br><br>Stelle deine Rangvergabe wie folgt dar (nenne nur die Antwort und den dazugehörigen Rang):<br><br>Studierendenantwort_X: Rang Y<br><br>Output:\n

Supplementary Table 18. Prompt $P_{v6.2}$ (minor spelling error) - Rank assessment

Du bist eine studdentische Hilfskraft für das Fach Makroökonomiek und bekommst eine Aufgabe gestelt. Ziel dieser Aufgaabe ist es, eine Rangfolge von Studierendenantworten auf eine offene Frge im Fach Makroökonmik basierend auf einer Musterlösung zu bilden.

Dies ist die offene Fage:

[INSERT_FRAGE]

Dies ist die Musterlösug:

[INSERT_SAMPLE_SOLUTION]

Dies sind fünf zufällig ausgewählte Studierendenantworten in zufäliger Reihenfolge:

Studierendenantwort_1: [STUDENT_ANSWER_1]

Studierendenantwort_2: [STUDENT_ANSWER_2]

Studierendenantwort_3: [STUDENT_ANSWER_3]

Studierendenantwort_4: [STUDENT_ANSWER_4]

Studierendenantwort_5: [STUDENT_ANSWER_5]

Deine Aufgabe ist es, für jede Studierendenatwort einen Rang zwischen 1 (beste Antwort) und 5 (schlechteste Antowrt) zu vergeben. Jeder Rang kann nur ein Mal vergeben werden. Bitte beachte bei der Vergabe der Ränge folgende Kriterien: Richtigkeit, Vollständigkiet und Relevanz für die Fragestellung. Dabei kannst Du deine Rangvergabe auf der jeweiligen Nähe der Studierendenatwort zur Musterlösung basieren. Falls Du zwei Antworten für gleichwertig hältst, musst Du dennoch eine bevorzugen. Ziehe Stil und Rechtschreibung als zusätzliche Entscheidungshilfen hinzu.

Stelle deine Rangvergabe wie folgt dar (nenne nur die Antwort und den dazugehörigen Rang):

Studierendenantwort_X: Rang Y

Output:\n

Supplementary Table 19. Prompt P_{v7} (suggestive and ambiguous instruction) - Rank assessment

Suppose you find yourself in the role of a student assistant for the subject of Macroeconomics and you are assigned an interesting, albeit somewhat challenging, task. Your goal? It involves creating a sort of ranking of student responses to an open-ended question in Macroeconomics, based on a given model answer.<br><br>Imagine the open question would be:<br><br>[INSERT_QUESTION]<br><br>And the model answer to it reads:<br><br>[INSERT_SAMPLE_SOLUTION]<br><br>Now, here are five randomly selected responses from students, in no specific order:<br><br>Student answer_1: [STUDENT_ANSWER_1]<br><br>Student answer_2: [STUDENT_ANSWER_2]<br><br>Student answer_3: [STUDENT_ANSWER_3]<br><br>Student answer_4: [STUDENT_ANSWER_4]<br><br>Student answer_5: [STUDENT_ANSWER_5]<br><br>Your task, or rather, your challenge, is to assign a rank to each of these responses between 1 (as the best answer) and 5 (as the least convincing answer). It is important that each rank remains unique. In assigning the ranks, you might consider the following criteria: the correctness of the answer, its completeness, and how relevant it is to the posed question. It could be helpful to base your decisions on how closely each answer matches the model solution. Should you find yourself considering two answers as equivalent, it would still be necessary to show a slight preference. Perhaps style and spelling could serve as additional factors in making a decision.<br><br>The way you present your ranking might look something like this (by only naming the answer and the corresponding rank):<br><br>Student answer_X: Rank Y<br><br>Output:\n

Supplementary Table 20. Prompt P_{v8} (role as a professor) - Rank assessment

You are a professor of Macroeconomics and have been given a task. The aim of this task is to rank student answers to an open question in macroeconomics based on a sample solution.

This is the open question:

[INSERT_QUESTION]

This is the sample solution:

[INSERT_SAMPLE_SOLUTION]

These are five randomly selected student answers in random order:

Student answer_1: [STUDENT_ANSWER_1]

Student answer_2: [STUDENT_ANSWER_2]

Student answer_3: [STUDENT_ANSWER_3]

Student answer_4: [STUDENT_ANSWER_4]

Student answer_5: [STUDENT_ANSWER_5]

Your task is to assign a rank between 1 (best answer) and 5 (worst answer) for each student answer. Each rank can only be awarded once. Please consider the following criteria when assigning ranks: Correctness, completeness and relevance to the question. You can base your ranking on the proximity of the student's answer to the sample solution. If you consider two answers to be of equal value, you must still give preference to one. Use style and spelling as additional decision-making criteria.

Present your ranking as follows (state only the answer and the corresponding rank):

Student answer_X: Rank Y

Output:\n

Supplementary Table 21. Prompt P_{v9} (role as an expert student assistant) - Rank assessment

You are an experienced student assistant for the subject of Macroeconomics and have been given a task. The aim of this task is to rank student answers to an open question in macroeconomics based on a sample solution.<br><br>This is the open question:<br><br>[INSERT_QUESTION]<br><br>This is the sample solution:<br><br>[INSERT_SAMPLE_SOLUTION]<br><br>These are five randomly selected student answers in random order:<br><br>Student answer_1: [STUDENT_ANSWER_1]<br><br>Student answer_2: [STUDENT_ANSWER_2]<br><br>Student answer_3: [STUDENT_ANSWER_3]<br><br>Student answer_4: [STUDENT_ANSWER_4]<br><br>Student answer_5: [STUDENT_ANSWER_5]<br><br>Your task is to assign a rank between 1 (best answer) and 5 (worst answer) for each student answer. Each rank can only be awarded once. Please consider the following criteria when assigning ranks: Correctness, completeness and relevance to the question. You can base your ranking on the proximity of the student's answer to the sample solution. If you consider two answers to be of equal value, you must still give preference to one. Use style and spelling as additional decision-making criteria.<br><br>Present your ranking as follows (state only the answer and the corresponding rank):<br><br>Student answer_X: Rank Y<br><br>Output:\n

Supplementary Table 22. Prompt P_{v10} (role as a non-expert student assistant) - Rank assessment

You are a beginner-level student assistant with limited knowledge in Macroeconomics and have been given a task. The aim of this task is to rank student answers to an open question in macroeconomics based on a sample solution.<br><br>This is the open question:<br><br>[INSERT_QUESTION]<br><br>This is the sample solution:<br><br>[INSERT_SAMPLE_SOLUTION]<br><br>These are five randomly selected student answers in random order:<br><br>Student answer_1: [STUDENT_ANSWER_1]<br><br>Student answer_2: [STUDENT_ANSWER_2]<br><br>Student answer_3: [STUDENT_ANSWER_3]<br><br>Student answer_4: [STUDENT_ANSWER_4]<br><br>Student answer_5: [STUDENT_ANSWER_5]<br><br>Your task is to assign a rank between 1 (best answer) and 5 (worst answer) for each student answer. Each rank can only be awarded once. Please consider the following criteria when assigning ranks: Correctness, completeness and relevance to the question. You can base your ranking on the proximity of the student's answer to the sample solution. If you consider two answers to be of equal value, you must still give preference to one. Use style and spelling as additional decision-making criteria.<br><br>Present your ranking as follows (state only the answer and the corresponding rank):<br><br>Student answer_X: Rank Y<br><br>Output:\n

Supplementary Table 23. Prompt P_{v11} (role not given) - Rank assessment

You are an assistant for the subject of Macroeconomics and have been given a task. The aim of this task is to rank student answers to an open question in macroeconomics based on a sample solution. <br>This is the open question: <br>[INSERT_QUESTION] <br>This is the sample solution: <br>[INSERT_SAMPLE_SOLUTION] <br>These are five randomly selected student answers in random order: <br>Student answer_1: [STUDENT_ANSWER_1] <br>Student answer_2: [STUDENT_ANSWER_2] <br>Student answer_3: [STUDENT_ANSWER_3] <br>Student answer_4: [STUDENT_ANSWER_4] <br>Student answer_5: [STUDENT_ANSWER_5] <br>Your task is to assign a rank between 1 (best answer) and 5 (worst answer) for each student answer. Each rank can only be awarded once. Please consider the following criteria when assigning ranks: Correctness, completeness and relevance to the question. You can base your ranking on the proximity of the student's answer to the sample solution. If you consider two answers to be of equal value, you must still give preference to one. Use style and spelling as additional decision-making criteria. <br>Present your ranking as follows (state only the answer and the corresponding rank): <br>Student answer_X: Rank Y <br>Output:\n

Supplementary Table 24. Prompt P_{v12} (advanced prompting: chain of thought) - Rank assessment

You are a student assistant for macroeconomics and are given a task. The aim of this task is to rank student answers to an open question in macroeconomics based on a sample solution.

This is the open question:

[INSERT_QUESTION]

This is the sample solution:

[INSERT_SAMPLE_SOLUTION]

These are five randomly selected student answers in random order:

Student answer_1: [STUDENT_ANSWER_1]

Student answer_2: [STUDENT_ANSWER_2]

Student answer_3: [STUDENT_ANSWER_3]

Student answer_4: [STUDENT_ANSWER_4]

Student answer_5: [STUDENT_ANSWER_5]

Your task is to assign a rank between 1 (best answer) and 5 (worst answer) for each student answer. Each rank can only be awarded once. Please consider the following criteria when assigning ranks: Correctness, completeness and relevance to the question. You can base your ranking on the proximity of the student's answer to the sample solution. If you consider two answers to be of equal value, you must still give preference to one. Use style and spelling as additional decision-making criteria.

Think step by step. You must explain your ranking, and then present your ranking allocation as follows (only name the answer and the corresponding rank):

Student answer_X: Rank Y

Output:\n

S9.2 Prompts for Point Assessment (English Translation)

Supplementary Table 25. Prompt P_{v2} (order of instruction) - Point assessment

You are a student assistant for macroeconomics and are given a task. The aim of this task is to award points for student answers to an open question in macroeconomics based on a sample solution. In the sample solution, the content for which a point is awarded is marked as follows: (1), (1 point) or (1P). The points (1), (1 point) or (1P) refer to the previous content.<br><br>Your task is to award a number of points for each student answer. 0 points are also possible. The sample solution contains all relevant and correct content for the question. It is complete. No more points can be awarded than in the sample solution.<br><br>You can base your award of points on the respective proximity of the student's answer to the points awarded for the content of the sample solution. This is the relevant content that is necessary to answer the question correctly and completely. A score equal to the number of points awarded by the sample solution means that the student's answer contains all the relevant content of the sample solution. Only whole points can be awarded. Add up the points you have awarded for each student answer. State only the total number of points for each student answer.<br><br>This is the open question:<br><br>[INSERT_QUESTION]<br><br>This is the sample solution:<br><br>[INSERT_SAMPLE_SOLUTION]<br><br>These are five randomly selected student answers in random order:<br><br>Student answer_1: [STUDENT_ANSWER_1]<br><br>Student answer_2: [STUDENT_ANSWER_2]<br><br>Student answer_3: [STUDENT_ANSWER_3]<br><br>Student answer_4: [STUDENT_ANSWER_4]<br><br>Student answer_5: [STUDENT_ANSWER_5]<br><br>Present your point assessment as follows (state only the answer and the number of points awarded):<br><br>Student answer_X: Points<br><br>Output:\n

Supplementary Table 26. Prompt P_{v3} (order of instruction) - Point assessment

This is the open question:

[INSERT_QUESTION]

This is the sample solution:

[INSERT_SAMPLE_SOLUTION]

These are five randomly selected student answers in random order:

Student answer_1: [STUDENT_ANSWER_1]

Student answer_2: [STUDENT_ANSWER_2]

Student answer_3: [STUDENT_ANSWER_3]

Student answer_4: [STUDENT_ANSWER_4]

Student answer_5: [STUDENT_ANSWER_5]

You are a student assistant for macroeconomics and are given a task. The aim of this task is to award points for student answers to an open question in macroeconomics based on a sample solution. In the sample solution, the content for which a point is awarded is marked as follows: (1), (1 point) or (1P). The points (1), (1 point) or (1P) refer to the previous content.

Your task is to award a number of points for each student answer. 0 points are also possible. The sample solution contains all relevant and correct content for the question. It is complete. No more points can be awarded than in the sample solution.

You can base your award of points on the respective proximity of the student's answer to the points awarded for the content of the sample solution. This is the relevant content that is necessary to answer the question correctly and completely. A score equal to the number of points awarded by the sample solution means that the student's answer contains all the relevant content of the sample solution. Only whole points can be awarded. Add up the points you have awarded for each student answer. State only the total number of points for each student answer.

Present your point assessment as follows (state only the answer and the number of points awarded):

Student answer_X: Points

Output:\n

Supplementary Table 27. Prompt P_{v4} (order of instruction) - Point assessment

You are a student assistant for macroeconomics and are given a task. The aim of this task is to award points for student answers to an open question in macroeconomics based on a sample solution. In the sample solution, the content for which a point is awarded is marked as follows: (1), (1 point) or (1P). The points (1), (1 point) or (1P) refer to the previous content.<br><br>This is the sample solution:<br><br>[INSERT_SAMPLE_SOLUTION]<br><br>This is the open question:<br><br>[INSERT_QUESTION]<br><br>These are five randomly selected student answers in random order:<br><br>Student answer_1: [STUDENT_ANSWER_1]<br><br>Student answer_2: [STUDENT_ANSWER_2]<br><br>Student answer_3: [STUDENT_ANSWER_3]<br><br>Student answer_4: [STUDENT_ANSWER_4]<br><br>Student answer_5: [STUDENT_ANSWER_5]<br><br>Your task is to award a number of points for each student answer. 0 points are also possible. The sample solution contains all relevant and correct content for the question. It is complete. No more points can be awarded than in the sample solution.<br><br>You can base your award of points on the respective proximity of the student's answer to the points awarded for the content of the sample solution. This is the relevant content that is necessary to answer the question correctly and completely. A score equal to the number of points awarded by the sample solution means that the student's answer contains all the relevant content of the sample solution. Only whole points can be awarded. Add up the points you have awarded for each student answer. State only the total number of points for each student answer.<br><br>Present your point assessment as follows (state only the answer and the number of points awarded):<br><br>Student answer_X: Points<br><br>Output:\n

Supplementary Table 28. Prompt P_{v5} (order of instruction) - Point assessment

You are a student assistant for macroeconomics and are given a task. The aim of this task is to award points for student answers to an open question in macroeconomics based on a sample solution. In the sample solution, the content for which a point is awarded is marked as follows: (1), (1 point) or (1P). The points (1), (1 point) or (1P) refer to the previous content.<br><br>This is the open question:<br><br>[INSERT_QUESTION]<br><br>These are five randomly selected student answers in random order:<br><br>Student answer_1: [STUDENT_ANSWER_1]<br><br>Student answer_2: [STUDENT_ANSWER_2]<br><br>Student answer_3: [STUDENT_ANSWER_3]<br><br>Student answer_4: [STUDENT_ANSWER_4]<br><br>Student answer_5: [STUDENT_ANSWER_5]<br><br>This is the sample solution:<br><br>[INSERT_SAMPLE_SOLUTION]<br><br>Your task is to award a number of points for each student answer. 0 points are also possible. The sample solution contains all relevant and correct content for the question. It is complete. No more points can be awarded than in the sample solution.<br><br>You can base your award of points on the respective proximity of the student's answer to the points awarded for the content of the sample solution. This is the relevant content that is necessary to answer the question correctly and completely. A score equal to the number of points awarded by the sample solution means that the student's answer contains all the relevant content of the sample solution. Only whole points can be awarded. Add up the points you have awarded for each student answer. State only the total number of points for each student answer.<br><br>Present your point assessment as follows (state only the answer and the number of points awarded):<br><br>Student answer_X: Points<br><br>Output:\n

Supplementary Table 29. Prompt $P_{v6.1}$ (major spelling error) - Point assessment

Du bhist eihne studäntishe Hilfskraft führ das Faach Makroökönomik unt bekommst aine Aufgahbe gestellt. Zihl deiser Aufgahbe is es, führ Studirendenantworten auf eine offähne Frage im Faach Makroökönomik basierent auf ainer Musterlösunk Punkte zu vergeben. In der Musterlösunk sünd diejenigen Inhalte wie folkt gekänzeichnet, führ die es ainen Punkt gibt: (1), (1 Punkd) odher (1P). Die Bepunktungen (1), (1 Punkd) oder (1P) beziähen sisch jewäils auf den vorausgegahnen Inhalt.<br><br>Dihes isst die offähne Frage:<br><br>[INSERT_FRAGE]<br><br>Dihes isst die Musterlösunk:<br><br>[INSERT_SAMPLE_SOLUTION]<br><br>Dihes sünd fünf zufällik ausgewählte Studirendenantworten in zufälliker Reihänfolge:<br><br>Studierendenantwort_1: [STUDENT_ANSWER_1]<br><br>Studierendenantwort_2: [STUDENT_ANSWER_2]<br><br>Studierendenantwort_3: [STUDENT_ANSWER_3]<br><br>Studierendenantwort_4: [STUDENT_ANSWER_4]<br><br>Studierendenantwort_5: [STUDENT_ANSWER_5]<br><br>Deine Aufgahbe isst es, für jede Studirendenantwort aine Punktzahl zu vergeben. Auch 0 Punkte sünd möglich. Die Musterlösunk enthält alle führ die Fragestellung relevante unt richtige Inhalte. Sie ist vollstängig. Es können nihct mehr Punkte vergeben wärden als in der Musterlösunk.<br><br>Dahbei kanst Du deine Vergabe der Punkte auf der jeweiligen Nähe der Studirendenantwort zu den bepunkteten Inhalten der Musterlösunk basieren. Dies sünd diejenigen relevanten Inhalte, die führ die inhaltlich korrekte unt vollständige Beantwortung der Frage notwendig sünd. Aine Punktzahl in Höhe der von der Musterlösunk vergebenen Punktzahl bedeuted, dass die Studirendenantwort alle relevanten Inhalte der Musterlösunk enthält. Es können nur ganze Punkte vergeben wärden. Addiere die jeweiligen Punkte, die du für aine Studirendenantwort vergeben hast. Gib für jede Studirendenantwort nur die jeweilige Gesamtpunktzahl an.<br><br>Stelle deine Punktevergabe wie folgt dar (nenne nur die Antwort unt die vergebene Punktzahl):<br><br>Studierendenantwort_X: Punktzahl<br><br>Output:\n

Supplementary Table 30. Prompt $P_{v6.2}$ (minor spelling error) - Point assessment

Du birst eine studentische Hilfskraft für das Fach Makroökonmie und bekommst eine Aufgabe gestelt. Ziel deiser Aufgabe ist es, für Studierendenantworten auf eine offene Frge im Fach Makroökonomik basirend auf einer Musterlösung Punkte zu vergebn. In der Musterlösug sind diejenigen Inhalte wie folgt gekenzeichnet, für die es einen Punk gibt: (1), (1 Punkt) oder (1P). Die Bepunktungen (1), (1 Punkt) oder (1P) beziehen sich jewils auf den vorausgehenden Inhalt.<br><br>Dies ist die offene Frge:<br><br>[INSERT_FRAGE]<br><br>Dies ist die Musterlösung:<br><br>[INSERT_SAMPLE_SOLUTION]<br><br>Dies sind fünf zufällig ausgewählte Studierendenantworten in zufälliger Reihenfolge:<br><br>Studierendenantwort_1: [STUDENT_ANSWER_1]<br><br>Studierendenantwort_2: [STUDENT_ANSWER_2]<br><br>Studierendenantwort_3: [STUDENT_ANSWER_3]<br><br>Studierendenantwort_4: [STUDENT_ANSWER_4]<br><br>Studierendenantwort_5: [STUDENT_ANSWER_5]<br><br>Deine Aufgabe ist es, für jede Studierendenantwort eine Punktzahl zu vergeben. Auch 0 Punkte sind möglich. Die Musterlösung enthält alle für die Fragestellung relevante und richtige Inhalte. Sie ist vollständig. Es können nicht mehr Punkte vergebn werden als in der Musterlösung.<br><br>Dabei kannst Du deine Vergabe der Punkte auf der jeweiligen Nähe der Studierendenantwort zu den bepunkteten Inhalten der Musterlösung basieren. Dies sind diejenigen relevanten Inhalte, die für die inhaltlich korrekte und vollständige Beantwortung der Frage notwendig sind. Eine Punktzahl in Höhe der von der Musterlösung vergebenen Punktzahl bedeutet, dass die Studierendenantwort alle relevanten Inhalte der Musterlösung enthält. Es können nur ganze Punkte vergebn werden. Addiere die jeweiligen Punkte, die du für eine Studierendenantwort vergebn hast. Gib für jede Studierendenantwort nur die jeweilige Gesamtpunktzahl an.<br><br>Stelle deine Punktevergabe wie folgt dar (nenne nur die Antwort und die vergebene Punktzahl):<br><br>Studierendenantwort_X: Punktzahl<br><br>Output:\n

Supplementary Table 31. Prompt P_{v7} (suggestive and ambiguous instruction) - Point assessment

Suppose you find yourself in the role of a student assistant for the subject of Macroeconomics and you are assigned a task. The goal of this task might be to assign points to student responses to an open question in Macroeconomics, based on a type of model solution. In this model solution, certain content might be marked in a specific way, for example with (1), (1 point), or (1P), which could indicate that one point can be assigned for these specific contents. These markings – (1), (1 point), or (1P) – might refer to the content immediately preceding them.<br><br>Let's imagine this is the open question:<br><br>[INSERT_QUESTION]<br><br>And this could be the model solution:<br><br>[INSERT_SAMPLE_SOLUTION]<br><br>Let's further assume there are five randomly selected student responses in random order:<br><br>Student answer_1: [STUDENT_ANSWER_1]<br><br>Student answer_2: [STUDENT_ANSWER_2]<br><br>Student answer_3: [STUDENT_ANSWER_3]<br><br>Student answer_4: [STUDENT_ANSWER_4]<br><br>Student answer_5: [STUDENT_ANSWER_5]<br><br>Your task could be to assign a score to each of these student responses, where even 0 points could be a possibility. The model solution could contain all the content relevant and correct for the question and be considered complete. It might be conceivable that no more points can be awarded than are provided for in the model solution.<br><br>You could base your allocation of points on how closely each student response matches the scored content of the model solution. These relevant contents could be those deemed necessary for a substantively correct and complete answer to the question. A score corresponding to the score given in the model solution could mean that the student response contains all the relevant contents of the model solution. It might be possible that only whole points can be awarded. You could be asked to add up the points you have assigned for each student response and to indicate only the total score for each student response.<br><br>Your ranking could then be presented like this (mention only the response and the score assigned):<br><br>Student answer_X: Points<br><br>Output:\n

Supplementary Table 32. Prompt P_{v8} (role as a professor) - Point assessment

You are a professor of Macroeconomics and have been given a task. The aim of this task is to award points for student answers to an open question in macroeconomics based on a sample solution. In the sample solution, the content for which a point is awarded is marked as follows: (1), (1 point) or (1P). The points (1), (1 point) or (1P) refer to the previous content.<br><br>This is the open question:<br><br>[INSERT_QUESTION]<br><br>This is the sample solution:<br><br>[INSERT_SAMPLE_SOLUTION]<br><br>These are five randomly selected student answers in random order:<br><br>Student answer_1: [STUDENT_ANSWER_1]<br><br>Student answer_2: [STUDENT_ANSWER_2]<br><br>Student answer_3: [STUDENT_ANSWER_3]<br><br>Student answer_4: [STUDENT_ANSWER_4]<br><br>Student answer_5: [STUDENT_ANSWER_5]<br><br>Your task is to award a number of points for each student answer. 0 points are also possible. The sample solution contains all relevant and correct content for the question. It is complete. No more points can be awarded than in the sample solution.<br><br>You can base your award of points on the respective proximity of the student's answer to the points awarded for the content of the sample solution. This is the relevant content that is necessary to answer the question correctly and completely. A score equal to the number of points awarded by the sample solution means that the student's answer contains all the relevant content of the sample solution. Only whole points can be awarded. Add up the points you have awarded for each student answer. State only the total number of points for each student answer.<br><br>Present your point assessment as follows (state only the answer and the number of points awarded):<br><br>Student answer_X: Points<br><br>Output:\n

Supplementary Table 33. Prompt P_{v9} (role as an expert student assistant) - Point assessment

You are an experienced student assistant for the subject of Macroeconomics and have been given a task. The aim of this task is to award points for student answers to an open question in macroeconomics based on a sample solution. In the sample solution, the content for which a point is awarded is marked as follows: (1), (1 point) or (1P). The points (1), (1 point) or (1P) refer to the previous content.<br><br>This is the open question:<br><br>[INSERT_QUESTION]<br><br>This is the sample solution:<br><br>[INSERT_SAMPLE_SOLUTION]<br><br>These are five randomly selected student answers in random order:<br><br>Student answer_1: [STUDENT_ANSWER_1]<br><br>Student answer_2: [STUDENT_ANSWER_2]<br><br>Student answer_3: [STUDENT_ANSWER_3]<br><br>Student answer_4: [STUDENT_ANSWER_4]<br><br>Student answer_5: [STUDENT_ANSWER_5]<br><br>Your task is to award a number of points for each student answer. 0 points are also possible. The sample solution contains all relevant and correct content for the question. It is complete. No more points can be awarded than in the sample solution.<br><br>You can base your award of points on the respective proximity of the student's answer to the points awarded for the content of the sample solution. This is the relevant content that is necessary to answer the question correctly and completely. A score equal to the number of points awarded by the sample solution means that the student's answer contains all the relevant content of the sample solution. Only whole points can be awarded. Add up the points you have awarded for each student answer. State only the total number of points for each student answer.<br><br>Present your point assessment as follows (state only the answer and the number of points awarded):<br><br>Student answer_X: Points<br><br>Output:\n

Supplementary Table 34. Prompt P_{v10} (role as a non-expert student assistant) - Point assessment

You are a beginner-level student assistant with limited knowledge in Macroeconomics and have been given a task. The aim of this task is to award points for student answers to an open question in macroeconomics based on a sample solution. In the sample solution, the content for which a point is awarded is marked as follows: (1), (1 point) or (1P). The points (1), (1 point) or (1P) refer to the previous content.<br><br>This is the open question:<br><br>[INSERT_QUESTION]<br><br>This is the sample solution:<br><br>[INSERT_SAMPLE_SOLUTION]<br><br>These are five randomly selected student answers in random order:<br><br>Student answer_1: [STUDENT_ANSWER_1]<br><br>Student answer_2: [STUDENT_ANSWER_2]<br><br>Student answer_3: [STUDENT_ANSWER_3]<br><br>Student answer_4: [STUDENT_ANSWER_4]<br><br>Student answer_5: [STUDENT_ANSWER_5]<br><br>Your task is to award a number of points for each student answer. 0 points are also possible. The sample solution contains all relevant and correct content for the question. It is complete. No more points can be awarded than in the sample solution.<br><br>You can base your award of points on the respective proximity of the student's answer to the points awarded for the content of the sample solution. This is the relevant content that is necessary to answer the question correctly and completely. A score equal to the number of points awarded by the sample solution means that the student's answer contains all the relevant content of the sample solution. Only whole points can be awarded. Add up the points you have awarded for each student answer. State only the total number of points for each student answer.<br><br>Present your point assessment as follows (state only the answer and the number of points awarded):<br><br>Student answer_X: Points<br><br>Output:\n

Supplementary Table 35. Prompt P_{v11} (role not given) - Point assessment

You are an assistant for the subject of Macroeconomics and have been given a task The aim of this task is to award points for student answers to an open question in macroeconomics based on a sample solution. In the sample solution, the content for which a point is awarded is marked as follows: (1), (1 point) or (1P). The points (1), (1 point) or (1P) refer to the previous content.

This is the open question:

[INSERT_QUESTION]

This is the sample solution:

[INSERT_SAMPLE_SOLUTION]

These are five randomly selected student answers in random order:

Student answer_1: [STUDENT_ANSWER_1]

Student answer_2: [STUDENT_ANSWER_2]

Student answer_3: [STUDENT_ANSWER_3]

Student answer_4: [STUDENT_ANSWER_4]

Student answer_5: [STUDENT_ANSWER_5]

Your task is to award a number of points for each student answer. 0 points are also possible. The sample solution contains all relevant and correct content for the question. It is complete. No more points can be awarded than in the sample solution.

You can base your award of points on the respective proximity of the student's answer to the points awarded for the content of the sample solution. This is the relevant content that is necessary to answer the question correctly and completely. A score equal to the number of points awarded by the sample solution means that the student's answer contains all the relevant content of the sample solution. Only whole points can be awarded. Add up the points you have awarded for each student answer. State only the total number of points for each student answer.

Present your point assessment as follows (state only the answer and the number of points awarded):

Student answer_X: Points

Output:\n

Supplementary Table 36. Prompt P_{v12} (advanced prompting: chain of thought) - Point assessment

You are a student assistant for macroeconomics and are given a task. The aim of this task is to award points for student answers to an open question in macroeconomics based on a sample solution. In the sample solution, the content for which a point is awarded is marked as follows: (1), (1 point) or (1P). The points (1), (1 point) or (1P) refer to the previous content.<br><br>This is the open question:<br><br>[INSERT_QUESTION]<br><br>This is the sample solution:<br><br>[INSERT_SAMPLE_SOLUTION]<br><br>These are five randomly selected student answers in random order:<br><br>Student answer_1: [STUDENT_ANSWER_1]<br><br>Student answer_2: [STUDENT_ANSWER_2]<br><br>Student answer_3: [STUDENT_ANSWER_3]<br><br>Student answer_4: [STUDENT_ANSWER_4]<br><br>Student answer_5: [STUDENT_ANSWER_5]<br><br>Your task is to award a number of points for each student answer. 0 points are also possible. The sample solution contains all relevant and correct content for the question. It is complete. No more points can be awarded than in the sample solution.<br><br>You can base your award of points on the respective proximity of the student's answer to the points awarded for the content of the sample solution. This is the relevant content that is necessary to answer the question correctly and completely. A score equal to the number of points awarded by the sample solution means that the student's answer contains all the relevant content of the sample solution. Only whole points can be awarded. Add up the points you have awarded for each student answer. State only the total number of points for each student answer.<br><br>Think step by step. You need to explain your points, and then present your ranking as follows (mention only the response and the score assigned):<br><br>Student answer_X: Points<br><br>Output:\n

S10 Robustness Checks and Extensions: Extended Results in Rank and Point Assessments

Supplementary Table 37. Summary statistics of IRR w.r.t prompt design effect using Kendall's W in rank assessment across all questions.

Prompt Version	Human team	AI-human team 1	AI-human team 2	AI-human team 3
P_{v1}: Original$_{\text{Initial run}}$	$0.792^{\pm 0.149}$	$0.789^{\pm 0.141}$	$0.806^{\pm 0.131}$	$0.787^{\pm 0.134}$
P_{v1}: Original$_{\text{Re-run}}$		$0.774^{\pm 0.176}$	$0.802^{\pm 0.154}$	$0.761^{\pm 0.175}$
P_{v2}: Structure-1	N/A	$0.776^{\pm 0.177}$	$0.796^{\pm 0.158}$	$0.762^{\pm 0.177}$
P_{v3}: Structure-2		$0.756^{\pm 0.177}$	$0.788^{\pm 0.162}$	$0.748^{\pm 0.174}$
P_{v4}: Structure-3		$0.761^{\pm 0.185}$	$0.788^{\pm 0.176}$	$0.742^{\pm 0.192}$
P_{v5}: Structure-4		$0.753^{\pm 0.167}$	$0.786^{\pm 0.159}$	$0.755^{\pm 0.155}$
$P_{v6.1}$: Major Spelling Error		$0.761^{\pm 0.171}$	$0.787^{\pm 0.162}$	$0.749^{\pm 0.172}$
$P_{v6.2}$: Minor Spelling Error		$0.769^{\pm 0.176}$	$0.797^{\pm 0.162}$	$0.759^{\pm 0.179}$
P_{v7}: Suggestive and ambiguous instruction		$0.765^{\pm 0.180}$	$0.797^{\pm 0.169}$	$0.754^{\pm 0.184}$
P_{v8}: Role $\rightarrow$ Professor		$0.767^{\pm 0.182}$	$0.797^{\pm 0.160}$	$0.756^{\pm 0.180}$
P_{v9}: Role $\rightarrow$ Expert student assistant		$0.774^{\pm 0.169}$	$0.801^{\pm 0.156}$	$0.762^{\pm 0.167}$
P_{v10}:Role $\rightarrow$ Non-expert student assistant		$0.774^{\pm 0.166}$	$0.804^{\pm 0.154}$	$0.763^{\pm 0.163}$
P_{v11}:Role $\rightarrow$ Not given		$0.770^{\pm 0.176}$	$0.798^{\pm 0.160}$	$0.760^{\pm 0.178}$
P_{v12}:Advanced Prompting $\rightarrow$ Chain of thought		$0.770^{\pm 0.181}$	$0.798^{\pm 0.165}$	$0.760^{\pm 0.177}$

Standard deviation provided as superscript

Supplementary Table 38. Summary statistics of IRR w.r.t prompt design effect using Cronbach's Alpha in point assessment across all questions for five answer scoring.

Prompt Version	Human team	AI-human team 1	AI-human team 2	AI-human team 3
P_{v1}: Original$_{\text{Initial run}}$	0.944 [0.932, 0.954]	0.933 [0.918, 0.945]	0.932 [0.917, 0.944]	0.933 [0.919, 0.945]
P_{v1}: Original$_{\text{Re-run}}$		0.930 [0.915, 0.943]	0.930 [0.915, 0.942]	0.928 [0.913, 0.941]
P_{v2}: Structure-1	N/A	0.927 [0.911, 0.940]	0.926 [0.910, 0.939]	0.926 [0.910, 0.939]
P_{v3}: Structure-2		0.921 [0.904, 0.935]	0.920 [0.903, 0.934]	0.920 [0.902, 0.934]
P_{v4}: Structure-3		0.928 [0.913, 0.941]	0.928 [0.912, 0.941]	0.924 [0.908, 0.938]
P_{v5}: Structure-4		0.920 [0.902, 0.934]	0.921 [0.904, 0.935]	0.918 [0.901, 0.933]
$P_{v6.1}$: Major Spelling Error		0.931 [0.916, 0.943]	0.930 [0.916, 0.943]	0.929 [0.914, 0.942]
$P_{v6.2}$: Minor Spelling Error		0.931 [0.916, 0.943]	0.931 [0.916, 0.944]	0.930 [0.916, 0.943]
P_{v7}: Suggestive and ambiguous instruction		0.927 [0.911, 0.940]	0.926 [0.910, 0.939]	0.926 [0.910, 0.940]
P_{v8}: Role $\rightarrow$ Professor		0.932 [0.917, 0.944]	0.933 [0.919, 0.945]	0.932 [0.918, 0.944]
P_{v9}: Role $\rightarrow$ Expert student assistant		0.930 [0.916, 0.943]	0.928 [0.913, 0.941]	0.929 [0.914, 0.942]
P_{v10}: Role $\rightarrow$ Non-expert student assistant		0.934 [0.920, 0.946]	0.934 [0.920, 0.946]	0.934 [0.920, 0.946]
P_{v11}:Role $\rightarrow$ Not given		0.930 [0.915, 0.943]	0.929 [0.914, 0.942]	0.928 [0.913, 0.941]
P_{v12}:Advanced Prompting $\rightarrow$ Chain of thought		0.921 [0.904, 0.935]	0.922 [0.906, 0.936]	0.918 [0.900, 0.932]

Confidence Interval at 95% provided in square brackets

Supplementary Table 39. Summary statistics of IRR w.r.t prompt design effect using Cronbach's Alpha in point assessment across all questions for single answer scoring.

Prompt Version	Human team	AI-human team 1	AI-human team 2	AI-human team 3
P_{v1}: Original$_{\text{Initial run}}$	0.944 [0.932, 0.954]	0.942 [0.929, 0.952]	0.941 [0.929, 0.952]	0.944 [0.932, 0.954]
P_{v1}: Original$_{\text{Re-run}}$		0.935 [0.922, 0.947]	0.936 [0.922, 0.948]	0.936 [0.922, 0.947]
P_{v2}: Structure-1	N/A	0.928 [0.912, 0.941]	0.929 [0.914, 0.942]	0.929 [0.914, 0.942]
P_{v3}: Structure-2		0.921 [0.904, 0.935]	0.923 [0.906, 0.937]	0.924 [0.908, 0.938]
P_{v4}: Structure-3		0.933 [0.919, 0.945]	0.936 [0.923, 0.948]	0.935 [0.921, 0.946]
P_{v5}: Structure-4		0.932 [0.917, 0.944]	0.935 [0.921, 0.946]	0.935 [0.921, 0.946]
$P_{v6.1}$: Major Spelling Error		0.927 [0.911, 0.940]	0.929 [0.914, 0.942]	0.931 [0.916, 0.943]
$P_{v6.2}$: Minor Spelling Error		0.928 [0.913, 0.941]	0.930 [0.916, 0.943]	0.930 [0.915, 0.943]
P_{v7}: Suggestive and ambiguous instruction		0.929 [0.914, 0.942]	0.928 [0.912, 0.941]	0.930 [0.915, 0.943]
P_{v8}: Role $\rightarrow$ Professor		0.935 [0.921, 0.947]	0.937 [0.924, 0.924]	0.937 [0.923, 0.948]
P_{v9}: Role $\rightarrow$ Expert student assistant		0.933 [0.918, 0.945]	0.934 [0.919, 0.946]	0.934 [0.920, 0.946]
P_{v10}: Role $\rightarrow$ Non-expert student assistant		0.933 [0.919, 0.945]	0.934 [0.920, 0.946]	0.935 [0.921, 0.947]
P_{v11}:Role $\rightarrow$ Not given		0.934 [0.920, 0.946]	0.937 [0.924, 0.949]	0.935 [0.921, 0.947]
P_{v12}:Advanced Prompting $\rightarrow$ Chain of thought		0.928 [0.913, 0.941]	0.931 [0.916, 0.943]	0.932 [0.918, 0.945]

Confidence Interval at 95% provided in square brackets
